# Novel Oxidovanadium Complexes with Redox-Active R-Mian and R-Bian Ligands: Synthesis, Structure, Redox and Catalytic Properties

**DOI:** 10.3390/molecules26185706

**Published:** 2021-09-21

**Authors:** Anton N. Lukoyanov, Iakov S. Fomenko, Marko I. Gongola, Lidia S. Shul’pina, Nikolay S. Ikonnikov, Georgiy B. Shul’pin, Sergey Y. Ketkov, Georgy K. Fukin, Roman V. Rumyantcev, Alexander S. Novikov, Vladimir A. Nadolinny, Maxim N. Sokolov, Artem L. Gushchin

**Affiliations:** 1 Razuvaev Institute of Organometallic Chemistry, Russian Academy of Sciences, Tropinina 49, 603950 Nizhny Novgorod, Russia; anton@iomc.ras.ru (A.N.L.); sketkov@iomc.ras.ru (S.Y.K.); gera@iomc.ras.ru (G.K.F.); romanrum@iomc.ras.ru (R.V.R.); 2 Nikolaev Institute of Inorganic Chemistry, Siberian Branch of Russian Academy of Sciences, 3 Acad. Lavrentiev Ave., 630090 Novosibirsk, Russia; fom1-93@mail.ru (I.S.F.); m.gongola@g.nsu.ru (M.I.G.); spectr@niic.nsc.ru (V.A.N.); caesar@niic.nsc.ru (M.N.S.); 3 Nesmeyanov Institute of Organoelement Compounds, Russian Academy of Sciences, ulitsa Vavilova, dom 28, 119991 Moscow, Russia; shulpina@ineos.ac.ru (L.S.S.); ikonns@ineos.ac.ru (N.S.I.); 4 Semenov Federal Research Center for Chemical Physics, Russian Academy of Sciences, ulitsa Kosygina 4, 119991 Moscow, Russia; gbsh@mail.ru; 5 Chemistry and Physics, Plekhanov Russian University of Economics, Stremyannyi pereulok, dom 36, 117997 Moscow, Russia; 6 Institute of Chemistry, Saint Petersburg State University, Universitetskaya Nab., 7/9, 199034 Saint Petersburg, Russia; a.s.novikov@spbu.ru

**Keywords:** oxidovanadium(IV), redox-active ligands, monoiminoacenaphthenone, acenaphthene-1,2-diimine, crystal structure, cyclic voltammetry, DFT, oxidation of alkanes and alcohols

## Abstract

A new monoiminoacenaphthenone 3,5-(CF_3_)_2_C_6_H_3_-mian (complex **2**) was synthesized and further exploited, along with the already known monoiminoacenaphthenone dpp-mian, to obtain oxidovanadium(IV) complexes [VOCl_2_(dpp-mian)(CH_3_CN)] (**3**) and [VOCl(3,5-(CF_3_)_2_C_6_H_3_-bian)(H_2_O)][VOCl_3_(3,5-(CF_3_)_2_C_6_H_3_-bian)]·2.85DME (**4**) from [VOCl_2_(CH_3_CN)_2_(H_2_O)] (**1**) or [VCl_3_(THF)_3_]. The structure of all compounds was determined using X-ray structural analysis. The vanadium atom in these structures has an octahedral coordination environment. Complex **4** has an unexpected structure. Firstly, it contains 3,5-(CF_3_)_2_C_6_H_3_-bian instead of 3,5-(CF_3_)_2_C_6_H_3_-mian. Secondly, it has a binuclear structure, in contrast to **3**, in which two oxovanadium parts are linked to each other through V=O···V interaction. This interaction is non-covalent in origin, according to DFT calculations. In structures **2** and **3**, non-covalent π-π staking interactions between acenaphthene moieties of the neighboring molecules (distances are 3.36–3.40 Å) with an estimated energy of 3 kcal/mol were also found. The redox properties of the obtained compounds were studied using cyclic voltammetry in solution. In all cases, the reduction processes initiated by the redox-active nature of the mian or bian ligand were identified. The paramagnetic nature of complexes **3** and **4** has been proven by EPR spectroscopy. Complexes **3** and **4** exhibited high catalytic activity in the oxidation of alkanes and alcohols with peroxides. The yields of products of cyclohexane oxidation were 43% (complex **3**) and 27% (complex **4**). Based on the data regarding the study of regio- and bond-selectivity, it was concluded that hydroxyl radicals play the most crucial role in the reaction. The initial products in the reactions with alkanes are alkyl hydroperoxides, which are easily reduced to their corresponding alcohols by the action of triphenylphosphine (PPh_3_). According to the DFT calculations, the difference in the catalytic activity of **3** and **4** is most likely associated with a different mechanism for the generation of ^●^OH radicals. For complex **4** with electron-withdrawing CF_3_ substituents at the diimine ligand, an alternative mechanism, different from Fenton’s and involving a redox-active ligand, is assumed.

## 1. Introduction

**Scheme 1:**
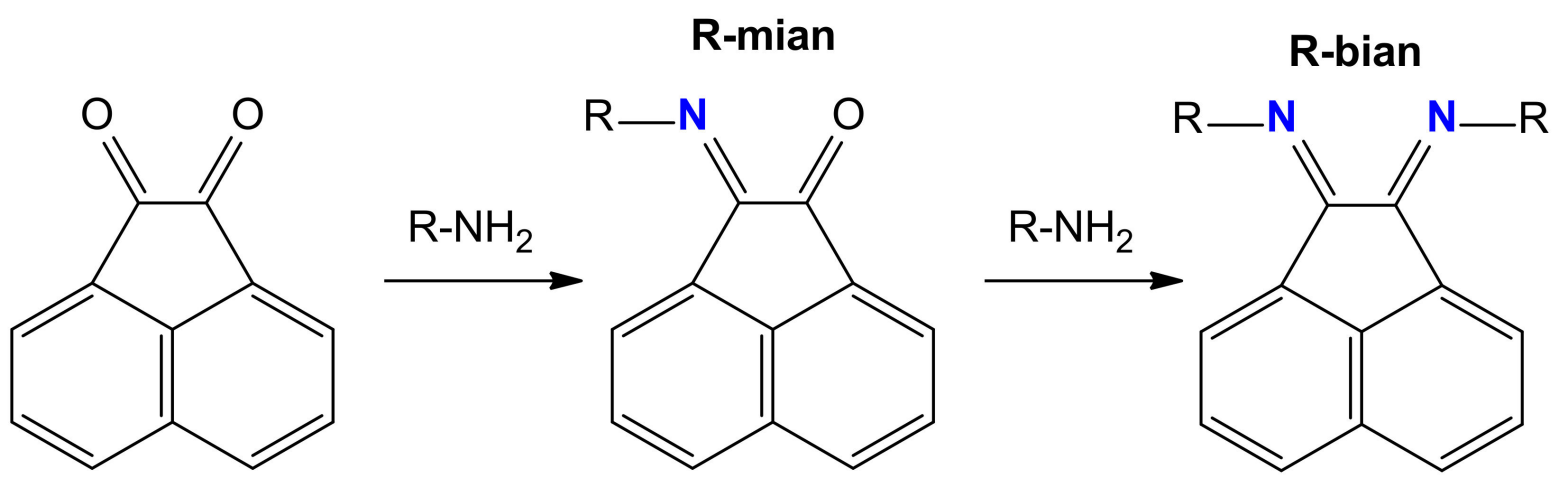
Scheme of synthesis of R-mian and R-bian ligands.

Redox-active ligands are capable of existing in several stable oxidation states, between which reversible redox changes are possible. They were introduced in the late 1960s and are now widely exploited by synthetics chemists to obtain a variety of metal complexes with unusual properties. Such metal complexes are often used to stimulate redox-based catalytic reactions and to activate small molecules [1,2,3,4,5,6,7,8,9,10]. The classical redox-active ligands include α-diimines, dithiolenes, and o-quinones. A unique property of these ligands lies in their ability to reversibly transform into radical anion or dianionic forms while maintaining a bond with a metal atom. Acenaphthene-1,2-diimine (R-bian) ligands (Scheme 1) are of particular note, as they are capable of accepting up to four electrons due to the reduction of both diimine and naphthalene components. Metal complexes with R-bian ligands are being actively studied in connection with the search for new effective catalysts for organic synthesis, as well as for materials with unusual redox and magnetic properties [11,12,13,14,15,16,17,18,19,20,21,22,23,24,25,26,27,28,29].

Monoiminoacenaphtheneones (R-mian) are condensation products between acenaphthenequinone and one mole of amine and contain both imine and carbonyl groups (Scheme 1). Coordinating ability and ability to be reduced of an R-mian is comparable to the corresponding R-bian [30,31]. The steric load on the metal is much lower in the case of R-mian, which simplifies ligand exchange near the metal center. However, the coordination properties of R-mian ligands have been studied extremely poorly and fragmentarily, in contrast to the R-bians [30,32].

The studies of the properties of transition metal complexes with R-mian ligands were initiated by a group under Prof. Tarun K. Panda [33,34,35,36]. In this series of works, the structural characteristics of the complexes were shown, and the catalytic activity of zinc derivatives in guanylation reactions of carbodiimides and isocyanates with anilines was discovered. Bromotricarbonyl complexes of manganese, based on p-Cl-C_6_H_4_-mian, turned out to be photosensitive and to release CO under visible light [37]. They can be classified as photoCORMs, which are sensitive to visible light even when in a solid state. A comparative study of ruthenium complexes, with R-mian and R-bian in the catalytic reactions of the epoxidation of alkenes [38,39] and oxidation of alcohols [38] was carried out in the works of Lahiri.

In this work, we report on a convenient preparative procedure for the synthesis of a new monoiminoacenaphtheneone (3,5-(CF_3_)_2_C_6_H_3_-mian (**2**)), as well as on the synthesis of new oxidovanadium(IV) complexes with R-mian (R = dpp) and R-bian (R = 3,5-(CF_3_)_2_C_6_H_3_)), which are rare examples of vanadium complexes with redox-active acanaphthene-imine ligands. Their structure, redox properties, and high catalytic activity in the oxidation of alkanes and alcohols with peroxides are discussed. DFT calculations were performed to explain the difference in the catalytic activity of new oxidovanadium complexes.

## 2. Results and Discussion

### 2.1. Synthesis of **1**–**4**

Monoiminoacenaphthenone 3,5-(CF_3_)_2_C_6_H_3_-mian (**2**) was not previously known; in this work, we describe a convenient preparative procedure for its synthesis from acenaphthenequinone and 3,5-trifluoromethylaniline.

**Scheme 2:**
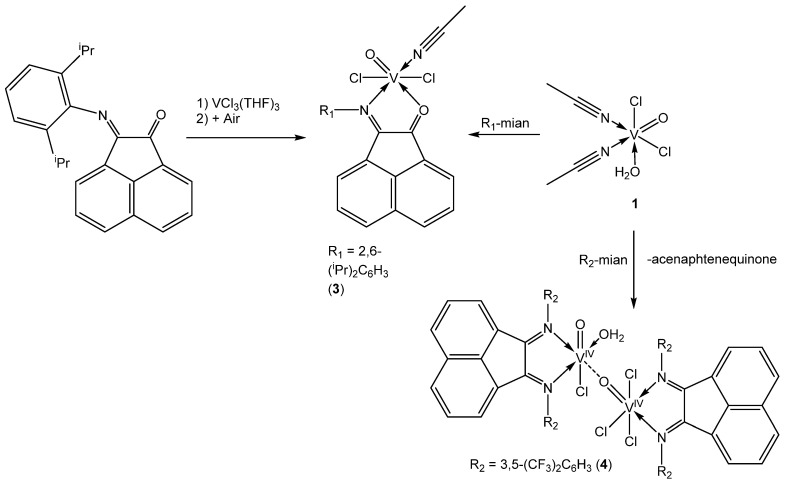
Scheme of the synthesis of complexes **3** and **4**.

Oxidovanadium(IV) complex **3** can be obtained in two ways. The first method (method A) is the oxidation of a mixture of VCl_3_(THF)_3_ and dpp-mian with atmospheric oxygen, where V(III) is oxidized to V(IV) with the formation of the {V=O}^2+^ fragment. The second method (method B) consists of using a pre-obtained complex V(IV), [VOCl_2_(CH_3_CN)_2_(H_2_O)] (**1**), synthesized from V_2_O_5_ in high yield (86%). It should be noted that the previously published procedure for the synthesis of complex **1** was less convenient, was a multi-step process, and required the use of less-available VOCl_2_ and toxic SOCl_2_ [40]. The crystal structure of **1** has been reported [41]. When **1** interacts with R-mian (dpp-mian or 3,5-(CF_3_)_2_C_6_H_3_-mian), labile H_2_O and CH_3_CN ligands are substituted (Scheme 2). By applying method B, the yield of complex **3** was increased from 21 to 83%. Nevertheless, method A was also successfully used to synthesize other oxidovanadium complexes with diimine ligands [20,42,43,44,45,46,47].

Surprisingly, a complex with 3,5-(CF_3_)_2_C_6_H_3_-bian (complex **4**) was isolated instead of the expected complex with 3,5-(CF_3_)_2_C_6_H_3_-mian, when [VOCl_2_(CH_3_CN)_2_(H_2_O)] (**1**) reacted with 3,5-(CF_3_)_2_C_6_H_3_-mian (**2**). This can be explained by the disproportionation of 3,5-(CF_3_)_2_C_6_H_3_-mian to 3,5-(CF_3_)_2_C_6_H_3_-bian and acenaphthenequinone under reaction conditions. Indeed, free acenaphthenequinone was observed as the second product of this reaction. The reaction mixture was separated by recrystallization from dimethoxyethane (DME). Unlike complex **3**, complex **4** has a dimeric structure due to the V=O···V interaction between two different oxidovanadium fragments. The non-covalent character of this interaction was established with DFT calculations (see DFT calculations section).

We assume that the dimeric structure **4** is unstable in solution and transforms into a mononuclear structure [VOCl_2_(3,5-(CF_3_)_2_C_6_H_3_-bian)(CH_3_CN)] (**4′**, in CH_3_CN solution) similar to [VOCl_2_(dpp-mian)(CH_3_CN)] (**3**). This is evidenced by the identity of the EPR spectra for **3** and **4**. In addition, complexes **3** and **4** exhibit similar redox behavior in solution.

### 2.2. IR Spectra of **1**–**4**

FT-IR spectrum of 3,5-(CF_3_)_2_C_6_H_3_-mian (**2**) reveals vibration bands of the CH(Ar) group at 3068 cm^−1^ and the C=O group at 1732 cm^−1^, as well as ν(C-C), ν(C-N) and ν(C-F) bands in the 1656–1030 cm^−1^ range. FT-IR spectra of **3** and **4** show typical vibration bands of the CH group in the region of 3068–2868 cm^−1^ (for **3**) and 3084 cm^−1^ (for **4**), as well as ν(C-C) and ν(C-N) bands in the 1622–1041 cm^−1^ (for **3**) and 1667–1022 cm^−1^ (for **4**) range of R-mian and R-bian ligands. The bands at 2326 and 2295 cm^−1^ for **3** were assigned to ν(C≡N) vibrations of the coordinated CH_3_CN molecule [41,48]. The vibrations of the C=O group appeared as a strong band at 1704 cm^−1^ for **3**. As expected, no vibration bands of the C≡N and C=O groups were observed for **4**. The ν(V=O) stretching mode appeared as strong bands at 997 cm^−1^ (for **3**), and at 989 and 978 cm^−1^ (for **4**). A vibration band at 895 cm^−1^ was also found for **4**, which is typical for V=O···V=O structures [45,49].

### 2.3. Crystal Structures of **2**–**5**

Single crystals of free 3,5-(CF_3_)_2_C_6_H_3_-mian (**2**), suitable for X-ray diffraction, were obtained from a concentrated acetone solution. For complexes [VOCl_2_(dpp-mian)(CH_3_CN)] (**3**) and [VOCl(3,5-(CF_3_)_2_C_6_H_3_-bian)(H_2_O)][VOCl_3_(3,5-(CF_3_)_2_C_6_H_3_-bian)]·2.85DME (**4**), single crystals were obtained from a concentrated solution in acetonitrile or dimethoxyethane (DME), respectively. The recrystallization of the complex [VOCl_2_(CH_3_CN)_2_(H_2_O)] (**1**) from DME yielded crystals of [VOCl_2_(CH_3_CN)(DME)] (**5**) that were suitable for X-ray structural analysis.

**Figure 1:**
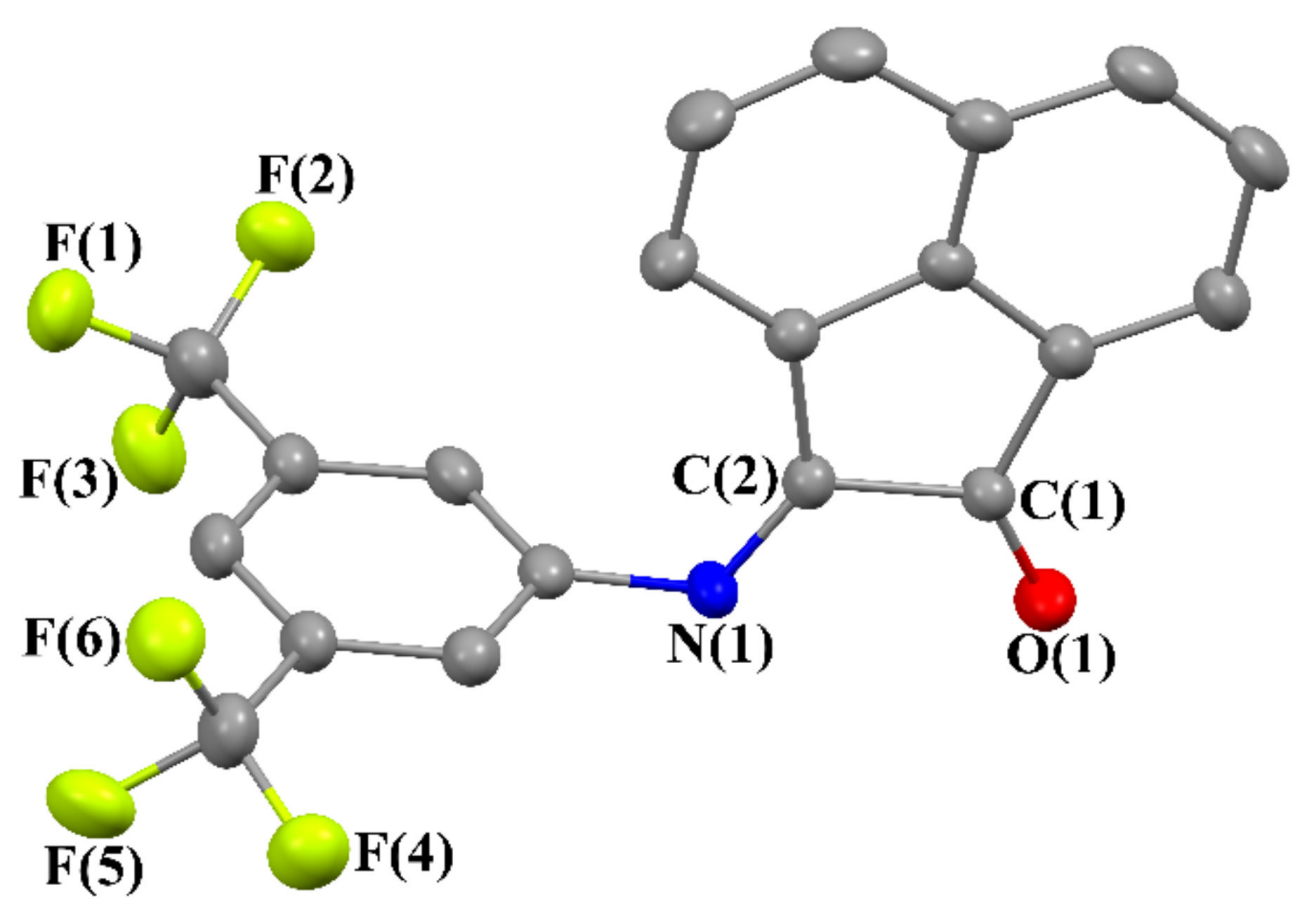
Molecular structure of compound **2**. The probability ellipsoids are drawn at the 30% level. The hydrogen atoms are omitted for clarity. Selected distances [Å] and angles [°]: O(1)-C(1) 1.204(3), N(1)-C(2) 1.269(3), N(1)-C(13) 1.420(3), C(1)-C(2) 1.546(3); C(2)-N(1)-C(13) 119.5(2), O(1)-C(1)-C(2) 125.0(2), N(1)-C(2)-C(1) 119.5(2).

According to the single-crystal X-ray analysis data, the compound 3,5-(CF_3_)_2_C_6_H_3_-mian (**2**) crystallizes in the P-1 space group. The molecular structure is shown in Figure 1.

**Figure 2:**
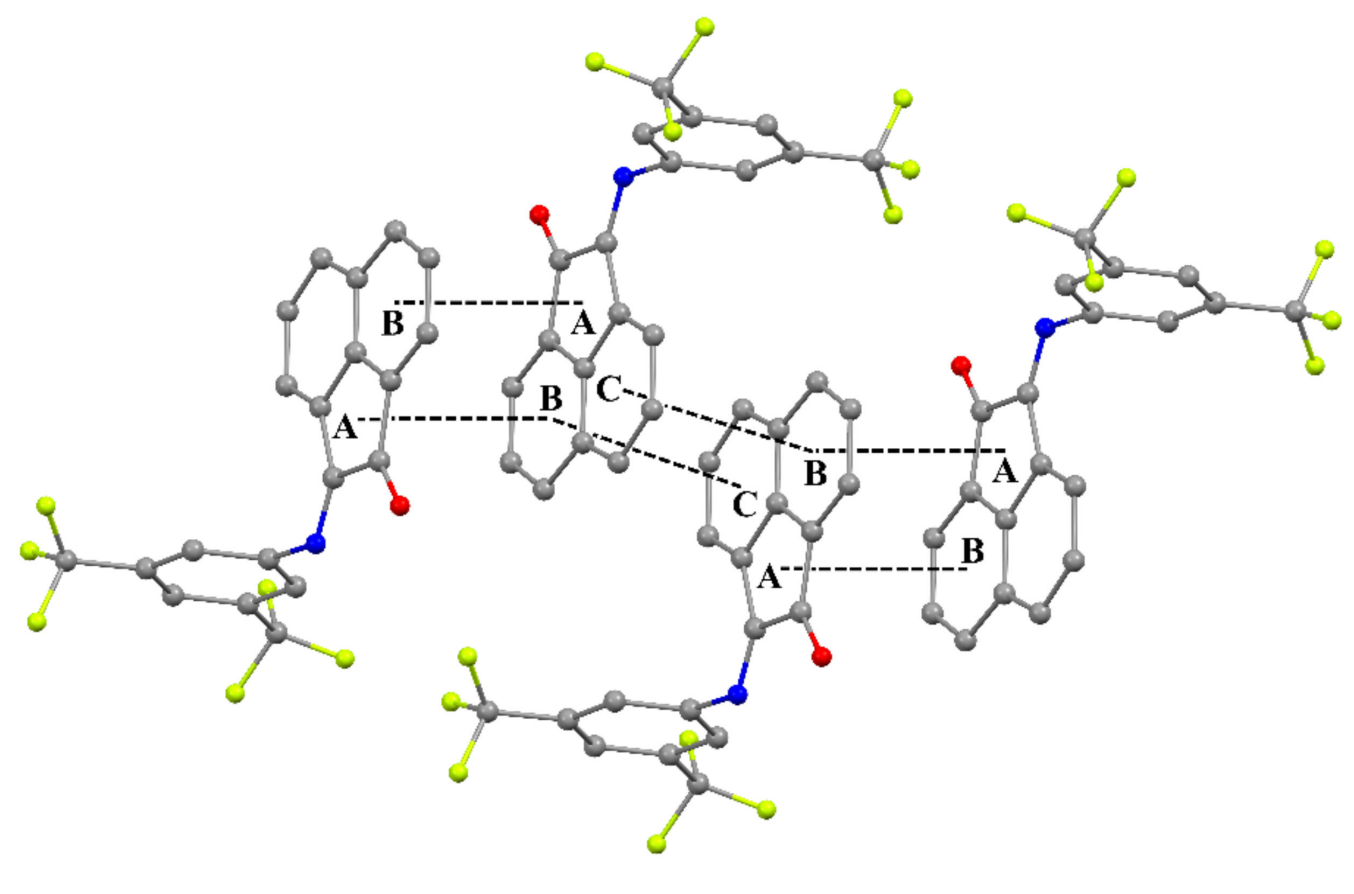
Fragment of the crystal packing of compound **2**. The hydrogen atoms are omitted for clarity.

The bond lengths C-N (1.269(3) Å), C-C (1.546(3) Å) and C-O (1.204(3) Å) unambiguously indicate the neutral state of the mian ligand with double C=N and C=O bonds and a single C-C bond [35,50,51,52]. The maximum deviation of atoms from the C_12_NO plane in **2** is 0.03 Å. The (CF_3_)_2_C_6_H_3_-ring is almost orthogonal to the acenaphthene π-system. The dihedral angle between these planes is 83.26°. In the crystal, the 3,5-(CF_3_)_2_C_6_H_3_-mian molecules are arranged in such a way that the acenaphthene plane of one molecule is parallel to and only slightly shifted with respect to the acenaphthene plane of the neighboring molecule. As a result, the **A**-**B** and **B**-**C** distances between the centers of the aromatic systems of neighboring molecules are 3.588 and 3.819 Å, respectively (Figure 2). This arrangement attests to the presence of π—π interactions between the molecules [53].

According to the single-crystal X-ray analysis data, there are two independent molecules (**A** and **B**) of **3** in the asymmetric unit cell. Figure S1 (Supporting Information) illustrates that both structures perfectly overlap when inverted (RMSD = 0.2342 Å) [54].

**Figure 3:**
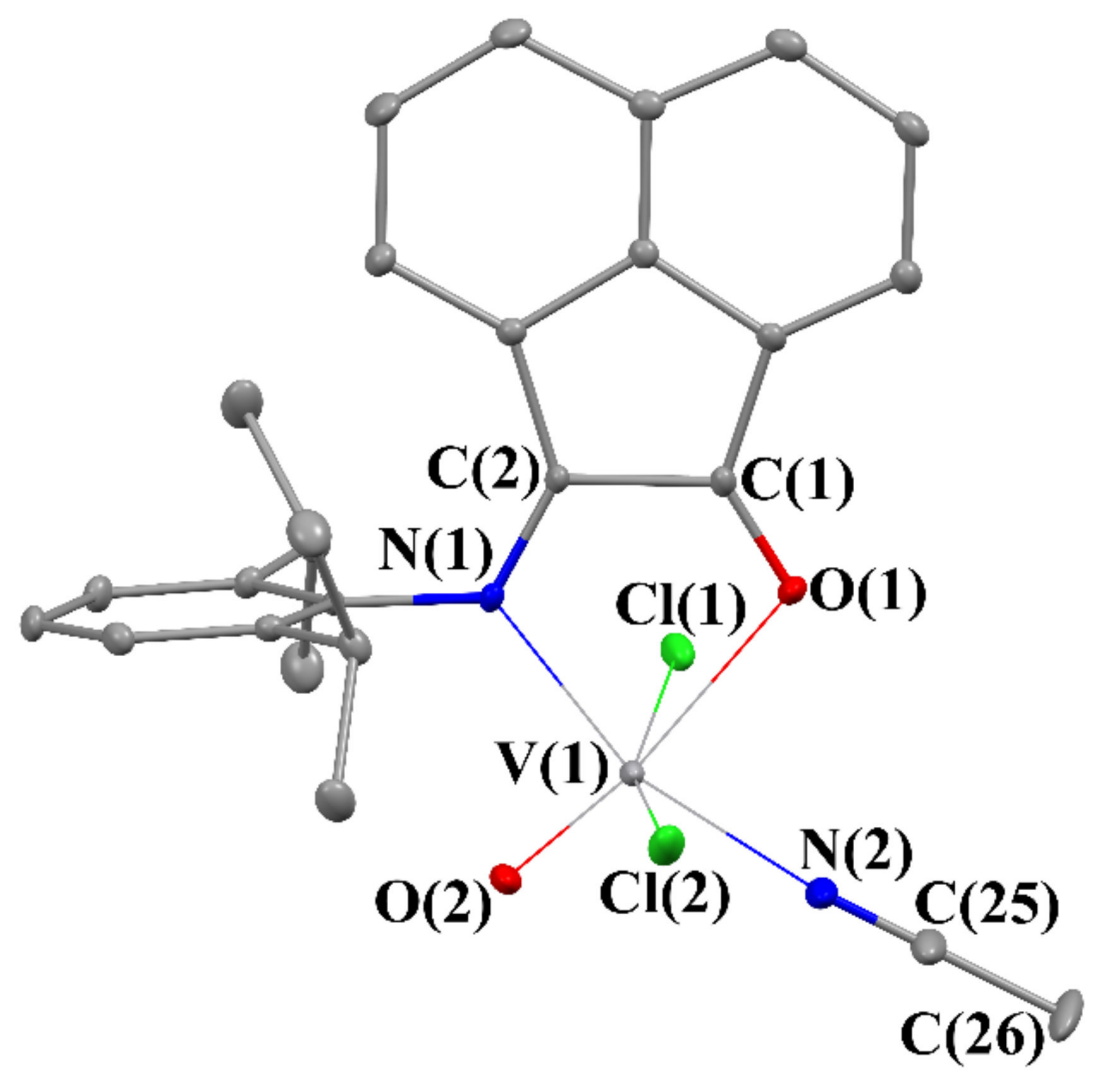
Molecular structure of compound **3**. The probability ellipsoids are drawn at the 30% level. The hydrogen atoms are omitted for clarity.

The vanadium in **3** has a distorted octahedral coordination environment, defined by the N and O atoms from dpp-mian, the O atom from the V=O group, and the N atom from the acetonitrile molecule, which define the equatorial plane. Two chlorine atoms occupy axial positions (Figure 3). It is interesting to note that in the related complex [VOCl_2_(2,6-Me_2_C_6_H_3_-bian)(H_2_O)] [55], the axial positions are occupied by oxygen atoms.

The bond lengths in the O-C-C-N fragment in **3** (Table S1) indicate the neutral state of dpp-mian in the complex [34,56]. The bond lengths V-Cl (2.3146(8)-2.3669(8) Å), V=O (1.585(2) Å), V-N (2.160(2)-2.163(2) Å) and V-N_CH3CN_ (2.107(3) Å) are characteristic of related vanadium complexes [20,41,43,45,47,57].

**Figure 4:**
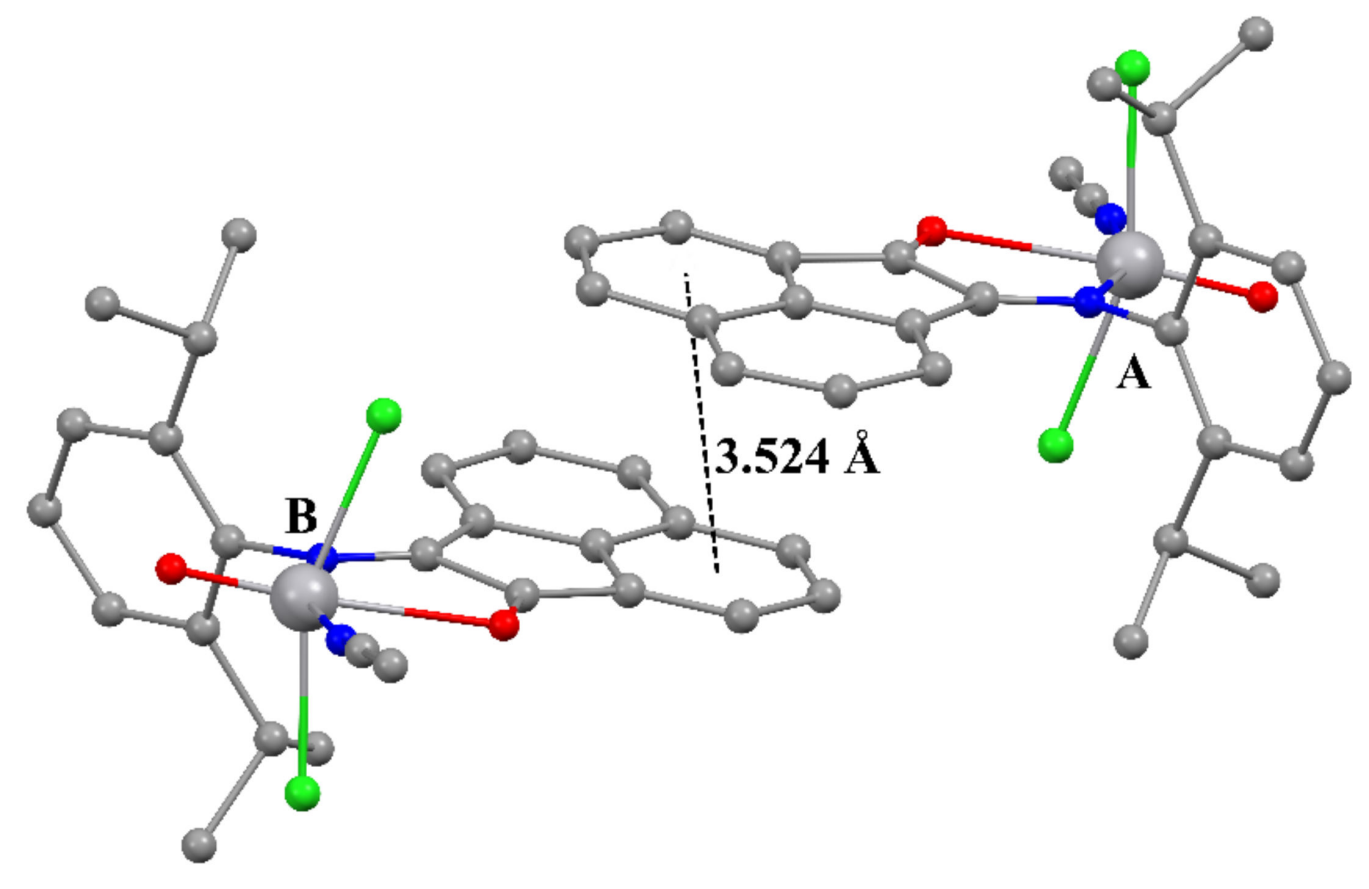
Fragment of crystal packing of complex **3**. The hydrogen atoms are omitted for clarity.

The molecules **A** and **B** in crystal **3** form dimeric pairs (Figure 4). The dihedral angle between the acenaphthene π-systems of neighboring molecules is 1.82°. The distance between the aromatic rings is 3.524 Å. These geometric characteristics indicate the presence of π-π interactions between the molecules [53].

**Figure 5:**
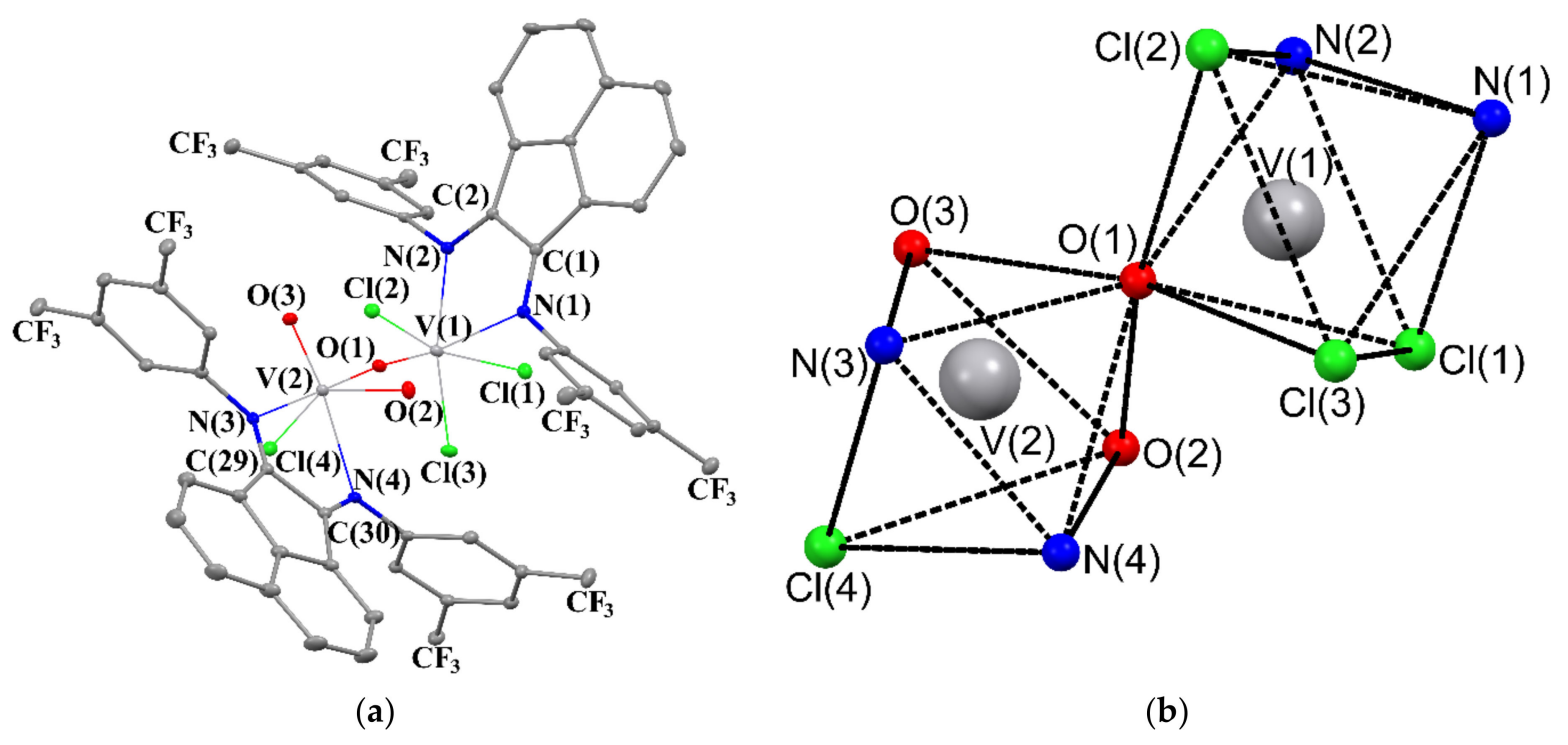
Molecular structure of complex **4** (**a**) and the coordination environment of vanadium atoms in **4** (**b**). The probability ellipsoids are drawn at the 30% level. The hydrogen and fluorine atoms are omitted for clarity.

According to the single crystal X-ray analysis data, two vanadium atoms in the complex **4** have a nonequivalent coordination environment (Figure 5a). Each vanadium atom is coordinated by a 3,5-(CF_3_)_2_C_6_H_3_-bian ligand. The distribution of bond lengths in the five-membered VNCCN metallacycles is in a narrow range and indicates the neutral form of the ligand [20,55]. The oxidovanadium moieties in **4** are bonded through a bridging oxygen atom, O(1). As a result, the distance V(1)=O(1) (1.6409(13) Å) increases in comparison with the V(2)=O(3) bond (1.5882(14) Å) (Table S2). The V(2)-O(1) distance in this dimeric V=O···V structure (2.0125(13) Å) is shorter than in polymeric [VO(L)X_2_]n (L = bpy, phen and X = Cl, Br) compounds (2.123(5)-2.125(5) Å) [45]. The vanadium atom V(1) is additionally bonded to three chloride anions. In turn, the V(2) atom is additionally bonded to one chloride anion and one water molecule. Thus, both vanadium atoms have the coordination number 6, and their coordination requirements are satisfied by two distorted octahedra with a common vertex (O(1) atom) (Figure 5b).

**Figure 6:**
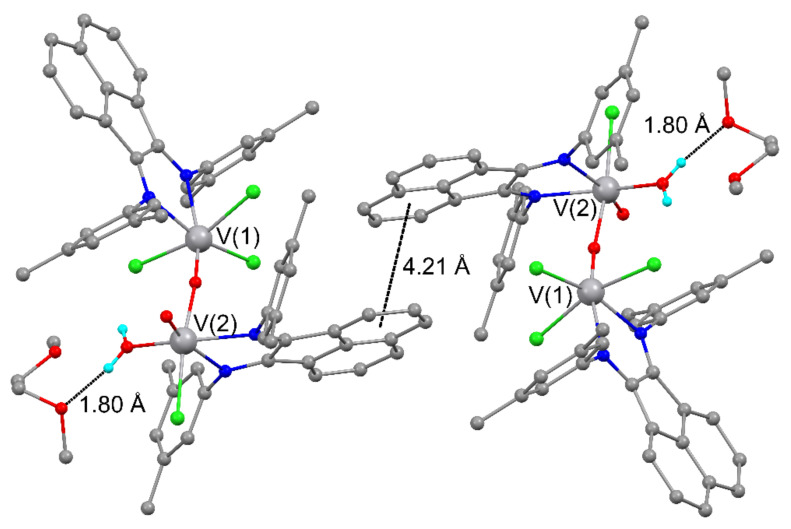
Fragment of the crystal packing of complex **4**. The hydrogen and fluorine atoms are omitted for clarity.

The molecules in crystal **4** are arranged in such a way that the bian ligand planes of neighboring molecules are parallel to each other, but displaced (Figure 6). The distances between the centers of the aromatic rings are 4.21 Å. These geometrical parameters significantly exceed the limit for intramolecular π···π interactions [53].

One of the uncoordinated dimethoxyethane molecules in crystal **4** is oriented to the hydrogen atoms of coordinated water (Figure 6). As a result, contracted intermolecular O-H···O contact (H···O 1.80 Å) is realized [58].

**Figure 7:**
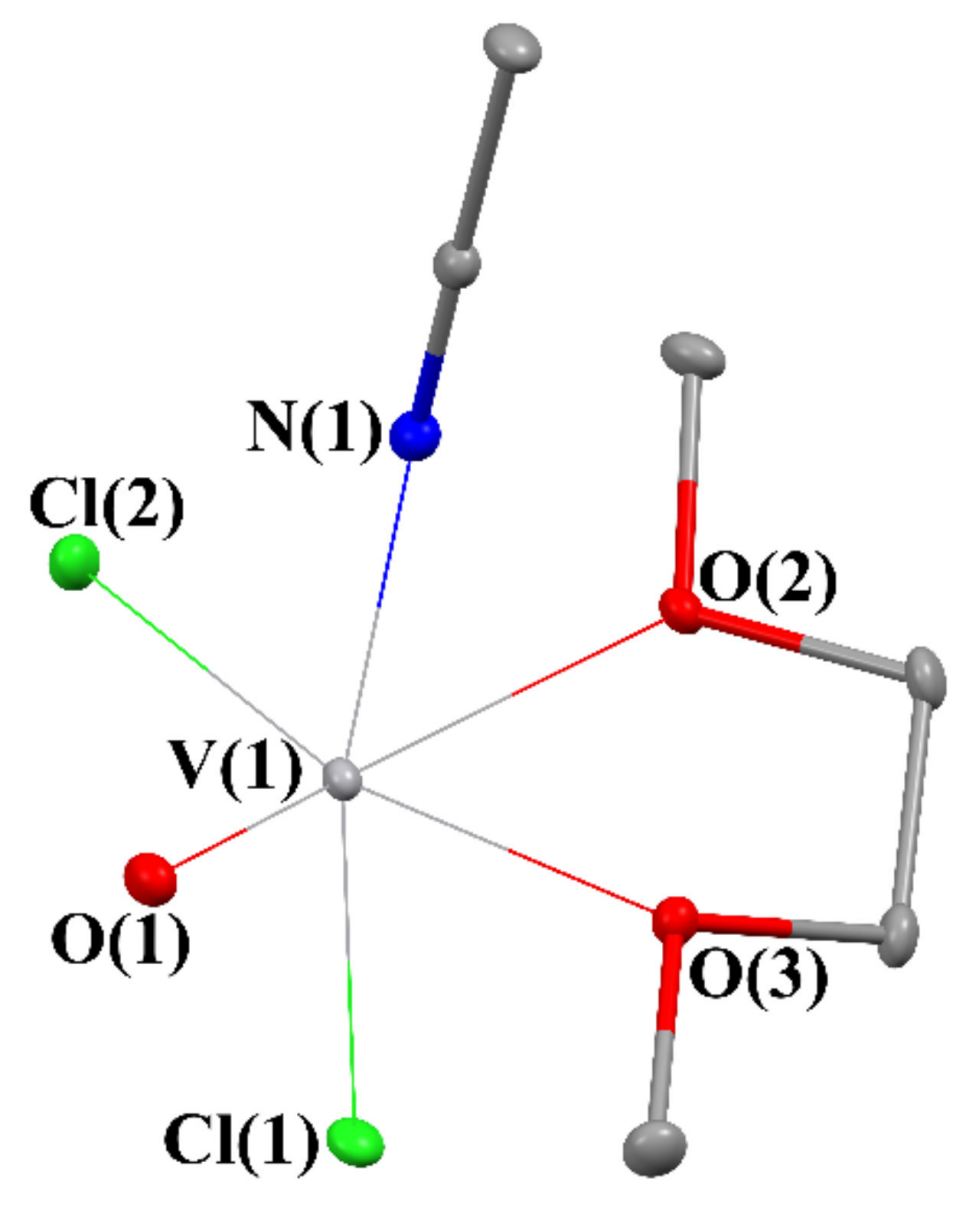
Molecular structure of compound **5**. The probability ellipsoids are drawn at the 30% level. The hydrogen atoms are omitted for clarity.

The vanadium atom in the structure of compound **5** has an octahedral environment similar to that observed in complex **3**. Instead of a neutral dpp-mian ligand, however, complex **5** contains a coordinated dimethoxyethane molecule (Figure 7). The axial positions are occupied by an acetonitrile molecule and a Cl atom. The DME molecule is asymmetrically coordinated in bidentate mode. The oxygen atom O(2), trans to V=O(1), has a longer V-O bond distance (2.2873(8) Å) than the cis oxygen atom O(3) (2.1112(8) Å), which is associated with the trans influence [47,59] The V=O, V-Cl and V-N_CH3CN_ distances in **5** are in good agreement with complex **3** (Table S1).

### 2.4. NMR Spectra of 3,5-(CF_3_)_2_C_6_H_3_-mian (**2**)

The doubling of signals in ^1^H and ^19^F NMR spectra was observed for 3,5-(CF_3_)_2_C_6_H_3_-mian (**2**) in various deuterated solvents (CDCl_3_, C_6_D_6_, thf-d8) (Figure S2), which is not typical for dpp-mian.

**Scheme 3:**
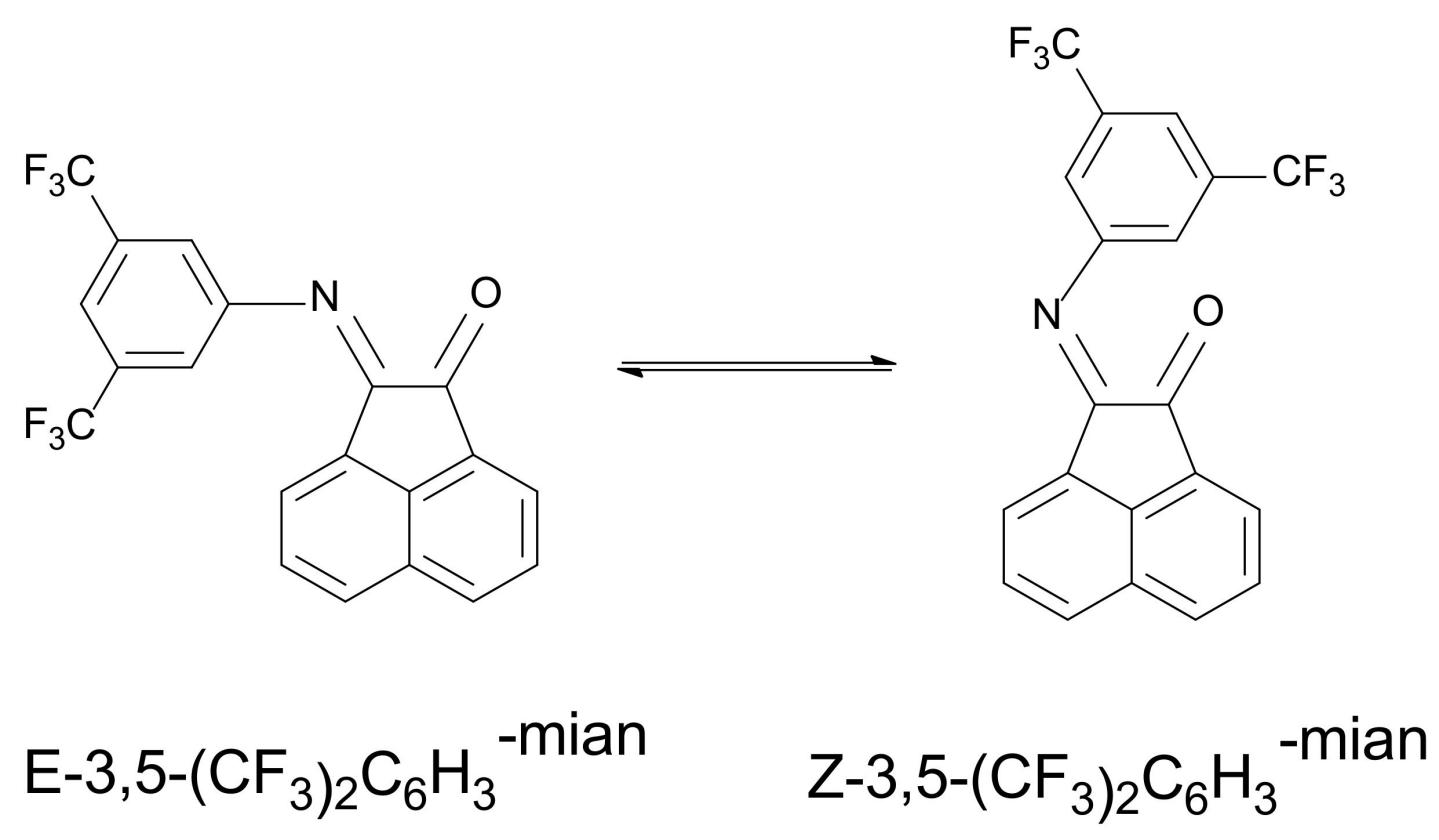
Isomerism of compound **2**.

This doubling of signals can be explained by the formation of Z and E isomers. Z-E isomerism for compound **2** in solution becomes possible due to the acceptor effect of CF_3_ groups on the C=N bond and the reduced bulkiness of 3,5-(CF_3_)_2_C_6_H_3,_ compared to 2,6-(^i^Pr)_2_C_6_H_3_ in dpp-mian (Scheme 3). In the crystal, only the E isomer has been found. The Z/E isomer ratio is solvent-dependent. Thus, in polar chloroform and tetrahydrofuran, the ratio is approximately 30/70, while in non-polar benzene it is 43/57. According to DFT calculations, the Z isomer is more stable by 0.3 kcal/mol in terms of the Gibbs free energy in the gas phase. 

### 2.5. Redox Properties of dpp-mian and **2**–**4**

Taking into account the pronounced π-acceptor properties of mian and bian ligands and their ability of multielectron reduction, the redox properties of the newly obtained compounds, **2–4,** were studied by cyclic voltammetry. For comparison, a cyclic voltammogram was recorded for dpp-mian under the same conditions. The data are summarized in Table 1. For dpp-mian and 3,5-(CF_3_)_2_C_6_H_3_-mian (**2**), two successive reduction processes were found at −1.16 (ΔE = 160 mV), −1.94 V, and at −0.85 (ΔE = 155 mV),−1.67 V (vs. Ag/AgCl), respectively. The first process is quasi-reversible, the second is irreversible, which reflects the limited stability of the reduced species (radical monoanion and dianion) in CH_2_Cl_2_ in the absence of the stabilizing effect of metal coordination. As expected, the electron-withdrawing CF_3_ substituents in **2** anodically shift the reduction potentials. For dpp-mian, an irreversible process is also observed at 1.64 V, which is most likely associated with the oxidation of dpp groups. Vanadium complexes **3** and **4** also exhibit several reduction processes. The first redox process at −1.23 V for **3** and −0.98 V for **4** is irreversible, probably due to the elimination of Cl^−^. This is supported by the fact that an anodic peak appears on the CV curve at about 1.1 V, which is typical for the oxidation of Cl^−^ [60,61]. The second process at −1.62 (ΔE = 130 mV) for **3** and −1.56 (ΔE = 110 mV) for **4** is quasi-reversible. For a similar complex [VOCl_2_(dpp-bian)], two quasi-reversible reduction processes were found at −0.32 and −1.05 V (vs Ag/AgCl) [20]. Taking into account the ability of both the oxidovanadium(IV) and mian moieties to be reduced, the observed redox processes can reflect a change in the oxidation state, both of the metal and the ligand (in the case of non-innocent behavior). In the positive region, an anodic process was found at 1.62 V for **3** and 1.52 V for **4**, which is likely related to the oxidation of V(IV). Irreversible oxidation of the [VOCl_2_(dpp-bian)] complex at 1.40 V was previously reported [20].

### 2.6. The EPR Spectra of **3** and **4**

**Figure 8:**
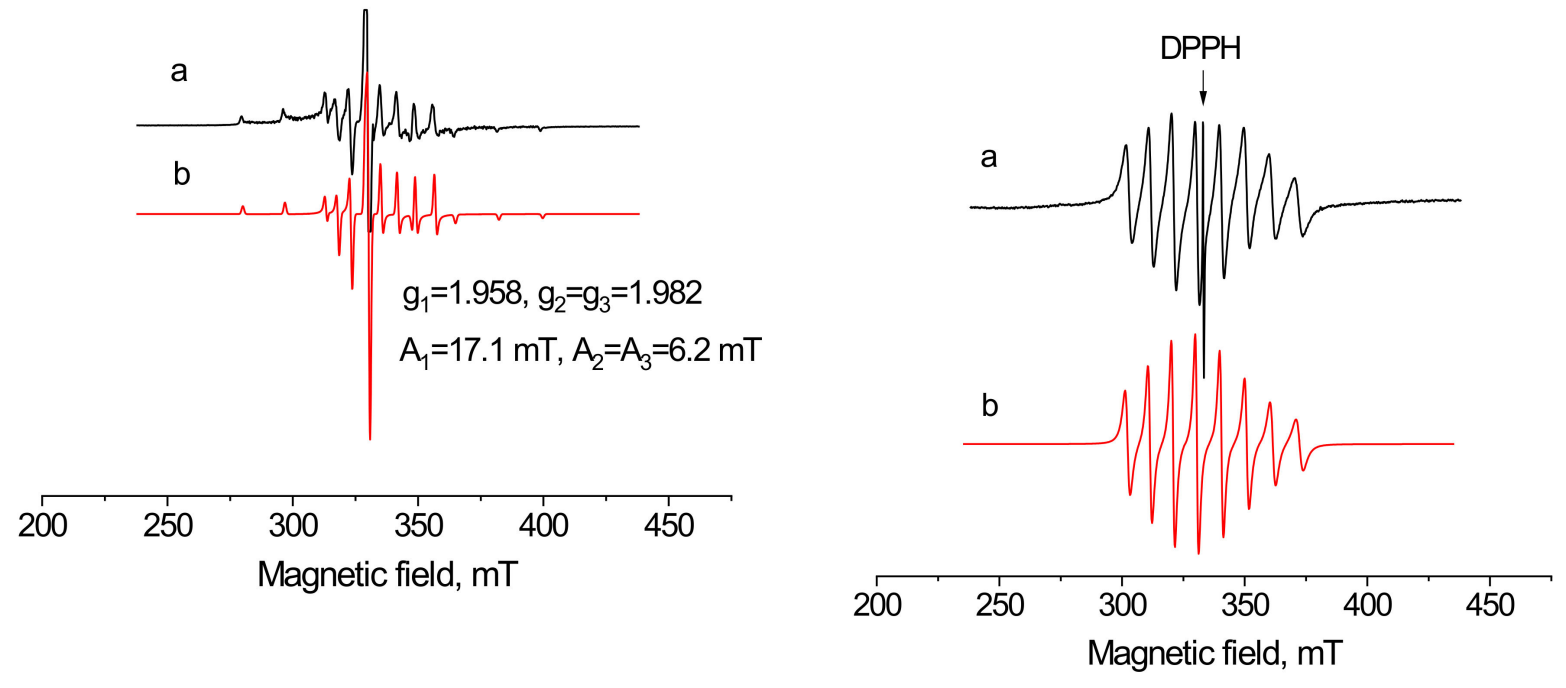
EPR spectra of **3** in CH_2_Cl_2_ at 77K (**left**) and 300K (**right**). a—Experimental, b—simulated. DPPH indicates the single EPR resonance of 2,2-diphenyl-1-picrylhydrazyl, which was used as an internal standard, with g = 2.0036.

**Figure 9:**
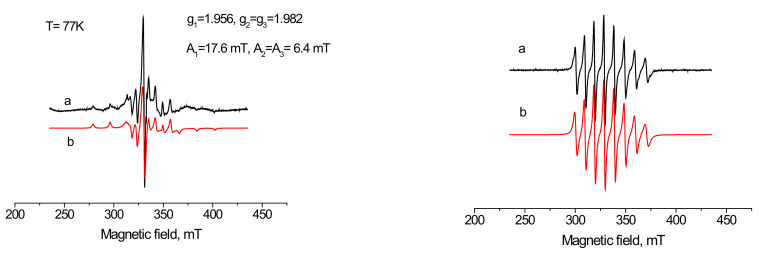
EPR spectra of **4** in CH_2_Cl_2_ at 77K (**left**) and 300K (**right**). a—Experimental, b—simulated.

The EPR spectra of **3** and **4** in CH_2_Cl_2_ at 77 and 300K are shown in Figure 8 and Figure 9. The spectra at 300K for both complexes present an eight-line isotropic signal, due to a hyperfine interaction with the ^51^V isotope (I = 7/2, 100%) which is characteristic for the V(IV) state (d^1^ electronic configuration). A simulation of the EPR spectra yielded the following g- and A-tensor values: g_1_ = 1.958, g_2_ = g_3_ = 1.982, the hyperfine interaction (HFI) tensor A_1_ = 17.1 mT, A_2_ = A_3_ = 6.2 mT for **3** and g_1_ = 1.956, g_2_ = g_3_ = 1.982, A_1_ = 17.6 mT, A_2_ = A_3_ = 6.4 mT for **4**. These values are typical for oxidovanadium(IV) complexes. EPR spectra at 300 K are very well simulated by the Easy Spin program with the obtained parameters at 77 K. The EPR parameters for **3** and **4** were almost identical to those for similar complexes: [VOCl_2_(dpp-bian)] (g_⊥_ = 1.972 and g_∥_ = 1.954, A_⊥_ = 6.3 mT, A_∥_ = 17.36 mT) [20] and [VOCl_2_(dbbpy)(H_2_O)] (g_⊥_ = 1.978, g_∥_ = 1.945, A_⊥_ = 6.5 mT, A_∥_ = 17.86 mT) [43].

### 2.7. DFT Calculations

**Figure 10:**
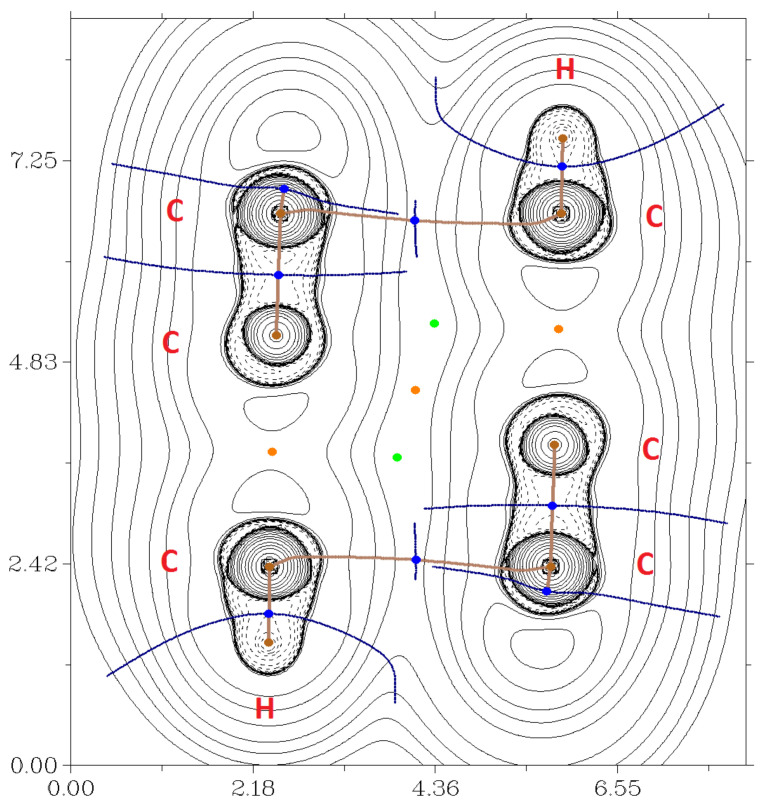
Contour line diagram of the Laplacian of electron density distribution ∇^2^ρ(**r**), bond paths, and selected zero-flux surfaces for intermolecular stacking interactions in the X-ray structure **2**. Bond-critical points (3, –1) are shown in blue, nuclear critical points (3, –3)—in pale brown, ring critical points (3, +1)—in orange, cage critical points (3, +3)—in light green, bond paths are shown as pale brown lines, length units—Å.

Inspection of the crystallographic data reveals the presence of intermolecular stacking interactions in the X-ray structures of 3,5-(CF_3_)_2_C_6_H_3_-mian (**2**) and [VOCl_2_(dpp-mian)(CH_3_CN)] (**3**). In order to confirm or disprove the existence of these noncovalent interactions in a solid state and quantify their strength from a theoretical viewpoint, the DFT calculations, followed by the topological analysis of the electron density distribution within the QTAIM approach [62], were carried out at the ωB97XD/6-31+G* level of theory for model structures (see Computational Details, Table S3 and attached xyz-files in Supporting Information). The results of the QTAIM analysis summarized in Table 2 (the Poincare–Hopf relationship was satisfied in both cases), the contour line diagram of the Laplacian of electron density distribution ∇^2^ρ(**r**), bond paths, and selected zero-flux surfaces for intermolecular stacking interactions in **2,** are shown in Figure 10 for illustrative purposes.

The QTAIM analysis of model structures demonstrates the presence of bond-critical points (3, –1) for intermolecular stacking interactions in the X-ray structures **2** and **3** (Table 2 and Figure 10). The low magnitude of the electron density (0.005–0.006 a.u.), positive values of the Laplacian of electron density (0.013–0.017 a.u.), and almost close to zero positive energy density (0.001 a.u.) in these bond-critical points (3, –1) are typical for stacking interactions in similar chemical systems [65,66,67,68,69]. The overall estimated strength of the intermolecular stacking interactions in X-ray structures **2** and **3** is almost the same in both cases (approx. 3 kcal/mol). The balance between the Lagrangian kinetic energy G(**r**) and potential energy density V(**r**) at the bond-critical points (3, –1) reveals no covalent contribution in all intermolecular contacts responsible for the stacking interactions in **2** and **3** (Table 2). The Laplacian of electron density is typically decomposed into the sum of contributions along the three principal axes of maximal variation, giving the three eigenvalues of the Hessian matrix (λ_1_, λ_2_ and λ_3_), and the sign of λ_2_ can be utilized to distinguish bonding (attractive, λ_2_ < 0) weak interactions from non-bonding ones (repulsive, λ_2_ > 0) [70,71]. Thus, the discussed intermolecular noncovalent interactions in **2** and **3** are slightly attractive (Table 2).

**Figure 11:**
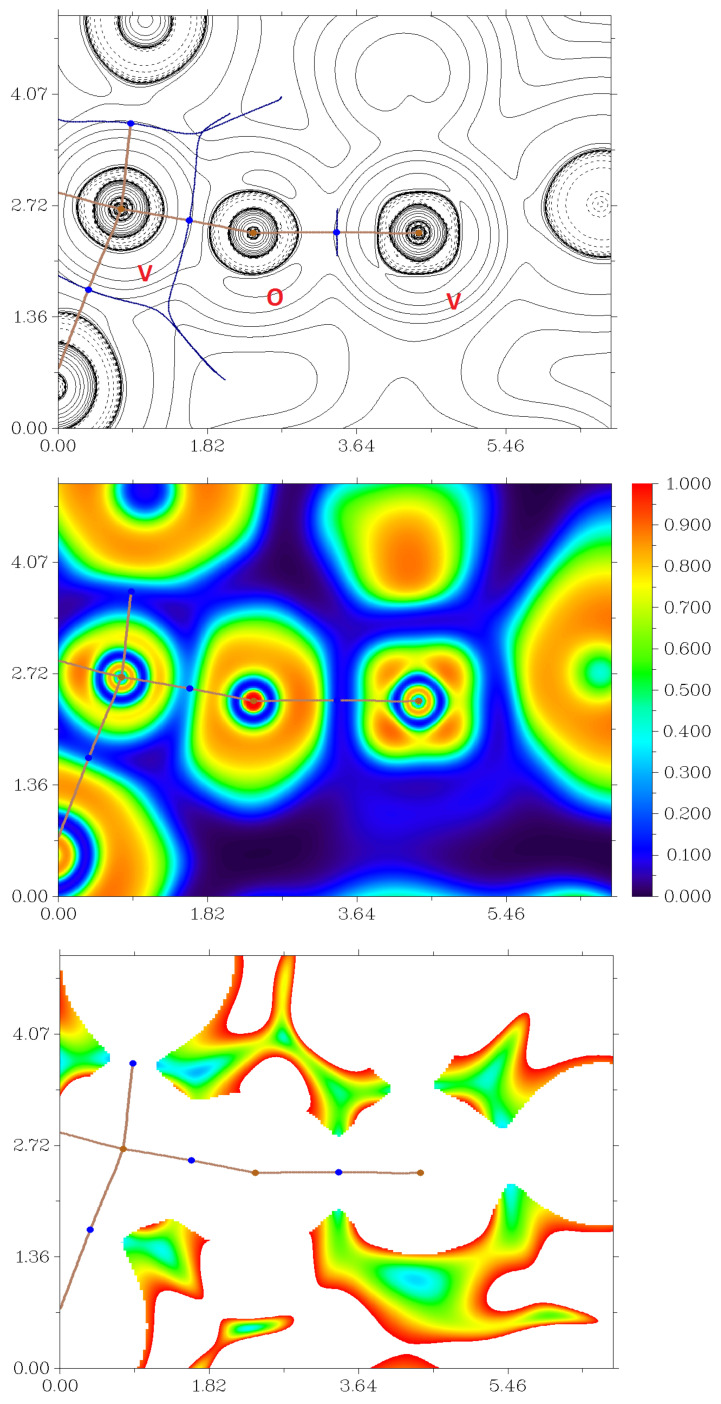
Contour line diagram of the Laplacian value of electron density distribution ∇^2^ρ(**r**), bond paths, and selected zero-flux surfaces (top panel), visualization of electron localization function (ELF, center panel), and reduced density gradient (RDG, bottom panel) analyses for coordination bond V–O (1.640 Å) and noncovalent contact V···O (2.013 Å) in the V–O···V moiety in X-ray structure **4**. Bond-critical points (3, –1) are shown in blue, nuclear-critical points (3, –3)—in pale brown, bond paths are shown as pale brown lines, length units—Å, and the color scale for the ELF and RDG maps is presented in a.u.

An inspection of the crystallographic data reveals the presence of two types of bonding between V and O atoms in the V=O···V moiety in the X-ray structure **4**, viz. a strong covalent bond V=O (1.640 Å) and noncovalent contact V···O (2.013 Å). In this case, also, the DFT calculations, followed by the topological analysis of the electron density distribution within the QTAIM approach [62], were carried out at the ωB97XD/6-31+G* level of theory for model structures (see Computational Details, Table S3 and attached xyz-file in Supporting Information). The results of the QTAIM analysis are summarized in Table 3, the contour line diagram of the Laplacian of electron density distribution ∇^2^ρ(r), bond paths, and selected zero-flux surfaces, the visualization of electron localization function (ELF), and reduced density gradient (RDG) analyses for coordination bond V–O and noncovalent contact V···O in the V–O···V moiety in the X-ray structure 4 are shown in Figure 11 for illustrative purposes. 

The QTAIM analysis of the model structure demonstrates the presence of bond-critical points (3, –1) for coordination bond V=O (1.640 Å) and noncovalent contact V···O (2.013 Å) in the V=O···V moiety in X-ray structure **4** (Table 3 and Figure 11). In the case of the short coordination bond V=O, the relatively high magnitude of the electron density, significantly positive value of the Laplacian of electron density, and a clearly negative energy density in the appropriate bond-critical point (3, –1) are typical for bonding interaction with a large covalent contribution, whereas the long-contact V···O is completely noncovalent (G(**r**)>|V(**r**)|). The sign of λ_2_ in bond-critical points (3, –1) can be utilized to distinguish bonding (attractive, λ_2_ < 0) interactions from non-bonding ones (repulsive, λ_2_ > 0) [70,71], and both discussed contacts between V and O atoms in the X-ray structure **4** are attractive (in case of coordination bond—in a greater extent, in case of noncovalent contact—in a lesser degree).

The presence of a redox-active ligand in catalysts **3** and **4,** and the possibility of its involvement in the mechanism of activation of hydrogen peroxide and generation of ^●^OH radicals for oxygenation of organic substrates, prompted us to carry out DFT calculations for possible intermediates. The distribution of HOMOs and LUMOs in hypothetical active species in cases of **3** and **4** (viz. species with H_2_O_2_ and ^●^OH ligands) are presented in Figures S3 and S4 in the Supporting Information. The HOMOs in both hypothetical active species [VOCl_2_(dpp-mian)(H_2_O_2_)] (**3**-H_2_O_2_) and [VOCl_2_(dpp-mian)(^●^OH)] (**3**-^●^OH) are located mainly on the V and Cl atoms, with the relatively small participation of dpp-mian and H_2_O_2_ or ^●^OH ligands and no contribution from the =O fragment. In the case of **3**-H_2_O_2_, the LUMO is completely localized on the V atom and dpp-mian ligand. In the case of **3**-^●^OH, the LUMO is completely localized on the V center, Cl, and ^●^OH ligands, without the participation of the dpp-mian ligand. The HOMO in [VOCl_2_(3,5-(CF_3_)_2_C_6_H_3_-bian)(H_2_O_2_)] (**4′**-H_2_O_2_) is located mainly on the V and Cl atoms, with the relatively small participation of H_2_O_2_ or 3,5-(CF_3_)_2_C_6_H_3_-bian ligands and no contribution from the =O fragment (very similar to **3**-H_2_O_2_). The LUMO in **4**-H_2_O_2_ is completely localized on the V atom and 3,5-(CF_3_)_2_C_6_H_3_-bian ligand, which is also similar to **3**-H_2_O_2_. Finally, the HOMO and LUMO in [VOCl_2_(3,5-(CF_3_)_2_C_6_H_3_-bian)(^●^OH)] (**4′**-^●^OH) are almost completely localized on the 3,5-(CF_3_)_2_C_6_H_3_-bian ligand, with a very small contribution from the V center. Note that HOMO–LUMO energy gaps in cases of **3**-H_2_O_2_ and **4′**-H_2_O_2_ are very close and relatively large (6.82 and 6.86 eV, respectively), whereas in the cases of **3**-^●^OH and **4′**-^●^OH, the HOMO–LUMO energy gaps are significantly different from each other and smaller (5.93 and 4.62 eV, respectively). The substantial difference of HOMO and LUMO shapes in **3**-^●^OH (frontier orbitals are not centered on the redox-active ligand) and **4′**-^●^OH (frontier orbitals are centered on the redox-active ligand) correlates well with their different catalytic activity toward the oxidation of alkanes and alcohols with peroxides.

### 2.8. Oxygenation of Alkanes and Alcohols

A system for the efficient oxidation of various organic compounds with hydrogen peroxide, based on a simple vanadate ion, was discovered in 1993. The obligatory component of this system is 2-pyrazine carboxylic acid (PCA) [72]. Later, this system was investigated in more detail, including the oxidation of alkanes, olefins, arenes, and alcohols with peroxides [73,74,75].

In this work, we report a study on the catalytic activity of the new oxidovanadium(IV) complexes **3** and **4,** as well as the effect of 2-pyrazine carboxylic acid on the activities of these complexes. We tried using nitric acid, but it turned out to be too aggressive and the catalysts quickly lost activity. Some of the authors have been working on the catalytic properties of vanadium complexes since 1993 and, during this time, have come to the conclusion that 2-pyrazine carboxylic acid is the most suitable co-catalyst for vanadium compounds [72,73,74,75,76,77].

**Figure 12:**
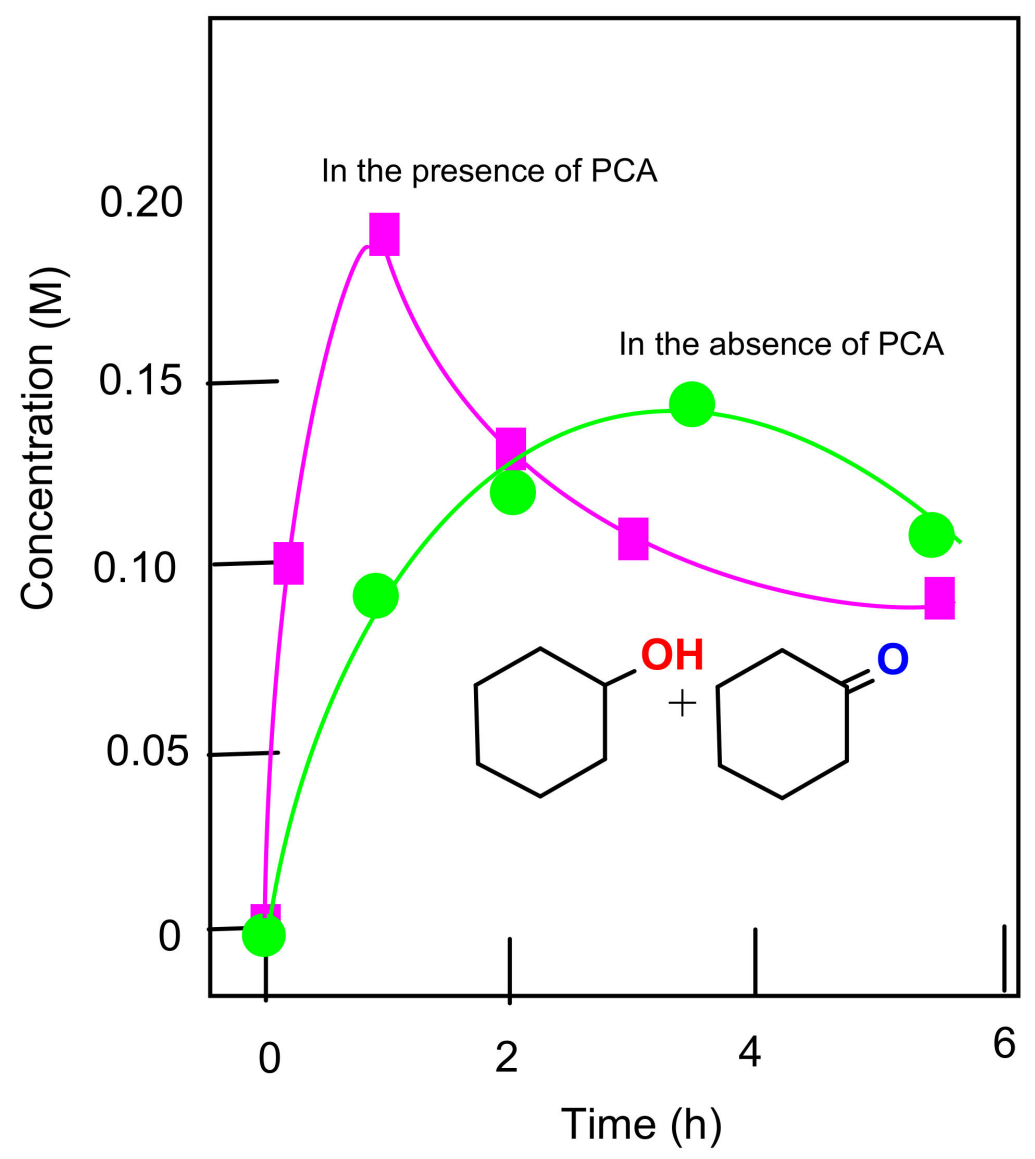
Accumulation of cyclohexanol and cyclohexanone (sum) in the oxidation of cyclohexane (0.46 M) with hydrogen peroxide (2.0 M, 50% aqueous) catalyzed by compound **3** (5 × 10^−4^ M) in the absence and in the presence of PCA (2 × 10^−3^ M) in MeCN at 50 °C. Concentrations of cyclohexanone and cyclohexanol were determined after the reduction of the aliquots with solid PPh_3_.

We have found that alkanes can be oxidized in acetonitrile solution into alkyl hydroperoxides with hydrogen peroxide in air, in the presence of a low concentration of complexes **3** or **4** (Figure S5 and Figure 12). The stability of the complexes toward the elimination of redox-active ligands in acetonitrile was confirmed using UV-vis and ^1^H NMR spectroscopies (Figures S6–S8). We studied the oxygenation of cyclic, linear and branched alkanes with systems **3** or **4**–H_2_O_2_, as well as **3** or **4**–PCA–H_2_O_2_ (PCA = 2-pyrazinecarboxylic acid) in acetonitrile solution under mild conditions (typical temperature is 50 °C). In the oxidation reaction of cyclohexane, in the presence of complex **3** as a catalyst, the total yield of cyclohexanol and cyclohexanone was 43% (TON = 400; the sum of the products divided by the amount of catalyst: [Mprod]/[cat]). For the reaction in the presence of catalyst **4**, the maximum yield of cyclohexanol and cyclohexanone was 27% (TON = 250). The addition of 2-pyrazine carboxylic acid to the reaction mixture of alkane oxidation increased the reaction rate (Figure 12).

**Figure 13:**
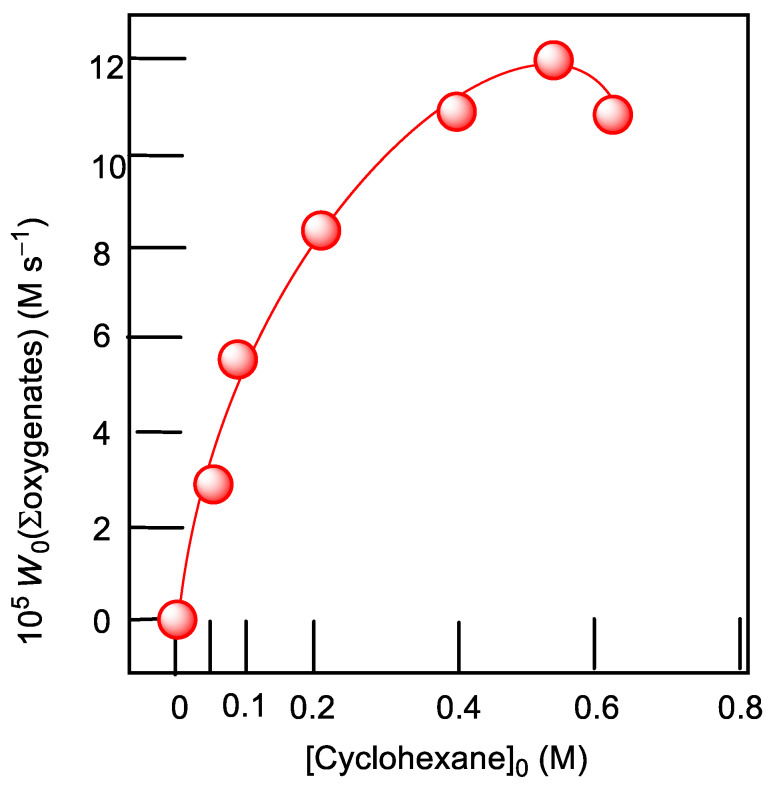
Dependence of the initial rate of oxygenate (sum cyclohexanol + cyclohexanone) formation *W*_0_ for complex **3** on the initial concentration of cyclohexane.

The dependence of the initial cyclohexane oxidation rate on the initial concentration of the substrate is shown in Figure 13. When the concentration of cyclohexane exceeds 0.55 M, the initial oxidation rate of the oxidation reaction does not depend on the initial concentration of the substrate.

Upon consideration of the regio- and bond-selectivity in the oxidation of n-heptane and methylcyclohexane, it is clear that that oxidation proceeds with the participation of free hydroxyl radicals. The data obtained are shown below.

The regio-selectivity parameters for the oxidation of n-heptane were obtained for complex **3:** C (1):C (2):C (3):C (4) = 1.0:27:2.7:2.4.

The bond-selectivity parameters for the oxidation of methylcyclohexane were also obtained for complex **3:** 1°:2°:3° = 1.0:5.2:14.0. These values correspond to the parameters obtained for oxidation reactions with the participation of hydroxyl radicals.

The results of the DFT calculations show that the mechanism of the formation of hydroxyl radicals in the case of catalyst **4**, apparently, differs from that usually accepted for Fenton-like systems. The redox-active 3,5-(CF_3_)_2_C_6_H_3_-bian ligand in the catalyst molecule, but not in the metal center, plays a decisive role in the formation of ^●^OH radicals from H_2_O_2_ and, therefore, in the oxidation of alkanes. We can assume a mechanism for the generation of ^●^OH radicals without the direct participation of the metal center, which was previously developed for catalysts based on transition and non-transition metals [78]. The activation and homolysis of hydrogen peroxide occur when it is coordinated to the metal center of the catalyst molecule and does not require a change in the oxidation state of the metal. This activation is associated with the redox nature of the 3,5-(CF_3_)_2_C_6_H_3_-bian ligand. Nevertheless, despite the redox-active nature of the dpp-mian ligand in catalyst **3**, in this case, the classical Fenton mechanism most likely takes place, in which the generation of ^●^OH radicals does not affect the change in the oxidation state of the redox-active ligand. The main difference between catalysts **3** and **4** is the different nature of the substituents in the aryl ring of the mono- or diimine ligand. In complex **3**, these are electron-donating i-Pr groups; in complex **4** they are electron-withdrawing CF_3_ groups.

**Figure 14:**
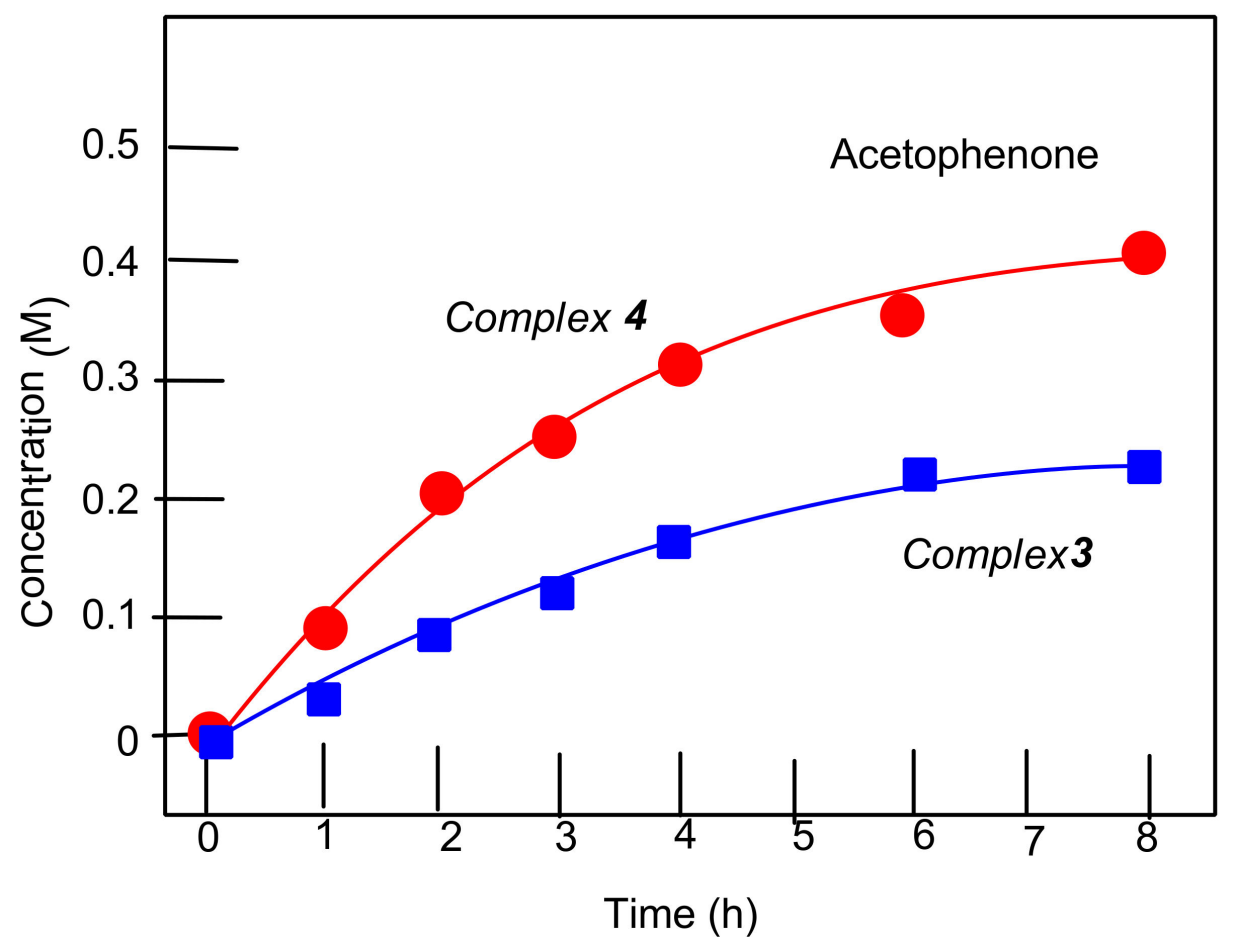
Accumulation of acetophenone in the oxidation of 1-phenyl ethanol (0.5 M) with tert-butyl hydroperoxide (1.5 M) catalyzed by complex **3** (5 × 10^−4^ M) (curve in blue) and by complex **4** (5 × 10^−4^ M) (curve in red) at 50 °C in acetonitrile. In order to quench the oxidation process concentrations of products were measured by GC only after the reduction of the reaction sample with solid PPh_3_.

**Figure 15:**
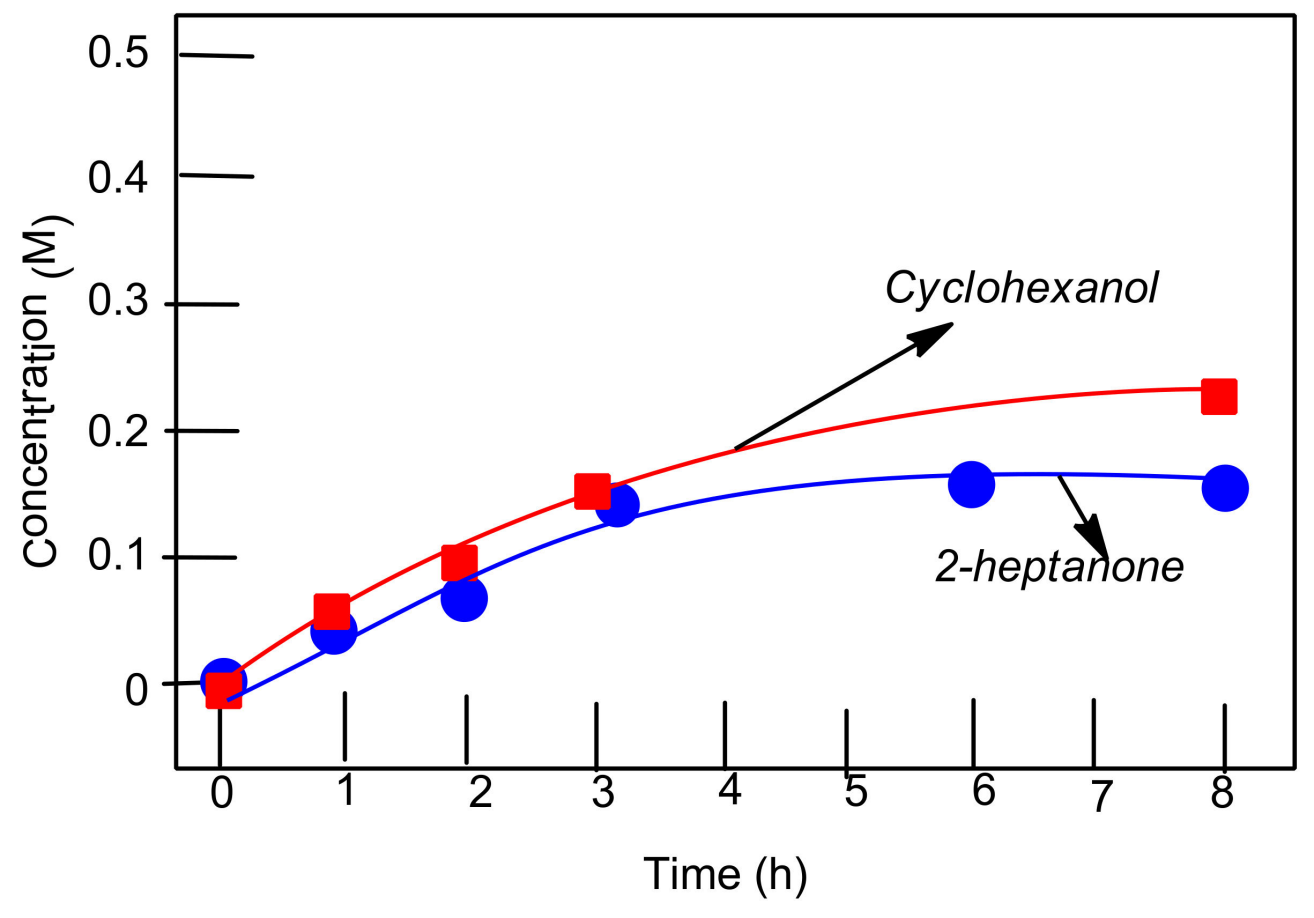
Accumulation of cyclohexanone in the oxidation of cyclohexanol (0.5 M) (curve in red) and 2-heptanone in the oxidation of 2-heptanol with tert-butyl hydroperoxide (1.5 M) (curve in blue) catalyzed by complex **3** (5 × 10^−4^ M) at 50 °C in acetonitrile. In order to quench the oxidation process, the concentrations of products were measured by GC only after the reduction of the reaction sample with solid PPh_3_.

A study on the oxidation of alcohols (phenyl ethanol, cyclohexanol and 2-heptanol) using tert-butyl hydroperoxide as an oxidizing agent and catalysts **3** and **4** was carried out (Figure 14 and Figure 15). Phenyl ethanol oxidized significantly better than the other alcohols. The addition of 2-pyrazine carboxylic acid to the reaction mixture did not affect the reaction rate or even slow down the reaction.

## 3. Conclusions

New oxidovanadium(IV) complexes **3** and **4,** with redox-active imino derivatives of acenaphthene, were obtained, the structural, redox and catalytic properties of which were studied in detail. In addition, a previously unknown monoiminoacenaphthenone 3,5-(CF_3_)_2_C_6_H_3_-mian (**2**), a new candidate for studying its coordination properties, was synthesized and characterized. Complex **4** has an unusual binuclear structure, due to the non-covalent V=O···V interaction between oxidovanadium moieties. To the best of our knowledge, complex **3** is the first example of a vanadium complex with monoiminoacenaphthenone (R-mian), and complex **4** is the first example of a vanadium complex with a 3,5-(CF_3_)_2_C_6_H_3_-bian ligand.

Complexes **3** and **4** exhibited high catalytic activity in the oxidation of alkanes with hydrogen peroxides under mild conditions. They are more active than the previously published catalyst [VOCl_2_(dpp-bian)] [20]. The addition of 2-pyrazine carboxylic acid to the reaction mixture of alkane oxidation leads to an increased reaction rate. Based on the data on the study of regio- and bond-selectivity, it was concluded that hydroxyl radicals play a crucial role in the reaction. The initial products in the reactions with alkanes are alkyl hydroperoxides, which are easily reduced to their corresponding alcohols by the action of triphenylphosphine (PPh_3_). Moreover, complexes **3** and **4** turned out to be good catalysts in the reactions of oxidation of alcohols to ketones with tert-butyl hydroperoxide as an oxidizing agent. The substantial difference of HOMO and LUMO shapes in [VOCl_2_(dpp-mian)(^●^OH)] (frontier orbitals are centered on the vanadium center) and [VOCl_2_(3,5-(CF_3_)_2_C_6_H_3_-bian)(^●^OH)] (frontier orbitals are centered on the redox-active ligand) intermediates correlates well with their different catalytic activity toward the oxidation of alkanes and alcohols with peroxides. We can assume an alternative mechanism for the generation of ^●^OH radicals in the case of complex **4,** with electron-withdrawing CF_3_ substituents at the bian ligand, which is different from the Fenton mechanism, with the involvement of a redox ligand.

## 4. Experimental Section

### 4.1. General Procedures

All manipulations were carried out in inert conditions using the standard Schlenk technique. The dpp-mian was synthesized according to the published procedure [31]. All solvents were distilled and degassed by standard methods before use.

### 4.2. Physical Measurements

Elemental C, H, and N analyses were performed with a EuroEA3000 Eurovector analyzer (EuroVector SpA, Milan, Italy). The IR spectra were recorded in the 4000–400 cm^–1^ range with a Perkin–Elmer System 2000 FTIR spectrometer, with samples in KBr pellets and Nujol. The cyclic voltammograms (CV) were recorded with a 797 VA Computrace system. All measurements were performed with a conventional three-electrode configuration consisting of glassy carbon working and platinum auxiliary electrodes and an Ag/AgCl/KCl reference electrode. The solvent was CH_2_Cl_2_ deoxygenated before use. Tetra-n-butylammonium hexafluorophosphate (0.1 M solution) was used as a supporting electrolyte. The concentration of the complex was approximately 10^−3^ M. The redox potential values (E_1/2_) were determined as (E_a_ + E_c_)/2, where E_a_ and E_c_ are anodic and cathodic peak potentials, respectively. EPR spectra were recorded in the X- and Q- bands at 77 and 300 K on an E-109 Varian spectrometer, equipped with an analog-to-digital signal converter. To analyze and simulate EPR spectra the EasySpin (Matlab software package) was used [79]. All measurements were taken with an external reference DPPH standard (2,2-diphenyl-1-picrylhydrazyl) for the correct determination of g–tensor values.

### 4.3. X-ray Data Collection and Structure Refinement

The X-ray diffraction data were collected on an Oxford Xcalibur Eos (compound **2**) and Bruker D8 Quest Photon II (complexes **3**–**5**) diffractometers (Mo-K_α_ radiation, ω-scan technique, λ = 0.71073 Å). The intensity data were integrated using the CrysAlisPro (**2**) [80] and SAINT (**3–5**) [81] programs. Absorption corrections were performed using the SADABS (**3–5**) program [82] and SCALE3 ABSPACK scaling algorithm (**2**) [83]. All structures were solved by a dual method [84] and refined on F_hkl_^2^ using the SHELXTL package [85]. All non-hydrogen atoms were refined anisotropically. All hydrogen atoms, except H(1) and H(2) in complex **4**, were placed in calculated positions and were refined in the riding model (U_iso_(H) = 1.5U_eq_(C) for CH_3_-groups and U_iso_(H) = 1.2U_eq_(C) for other groups). In turn, the H(1) and H(2) atoms in **4** were found from the Fourier syntheses of electron density and were refined isotropically. The asymmetric units of complex **4** contained 2.847 uncoordinated DME molecules per one molecule of the complex. The CF_3_-groups in compound **2** were disordered in three positions. Two CF_3_-groups were also disordered over two positions in **4**. To refine the disordered atoms, we used the EADP, ISOR, and DFIX instructions. Complex **3** was refined as a 2-component inversion twin. The absolute structure parameter is 0.379(16) [86].

The main crystallographic data and structure refinement details for **2–5** are presented in Table S4. The crystallographic data have been deposed in the Cambridge Crystallographic Data Centre under the deposition codes CCDC 2,093,694 (**2**), 2,093,695 (**3**), 2,093,696 (**4**) and 2,093,697 (**5**). These data can also be obtained free of charge at ccdc.cam.ac.uk/structures/from the Cambridge Crystallographic Data Centre.

### 4.4. Synthesis of [VOCl_2_(CH_3_CN)_2_(H_2_O)] (**1**)

To begin, 10.0 g of V_2_O_5_ was dissolved in 80 mL of concentrated hydrochloric acid. The resulting solution was evaporated by heating in air until a thick green slurry was formed. After cooling, the resulting solid residue was redissolved in acetonitrile (50 mL). As a result, a two-layer blue solution was formed. The top layer was drained and left for several days. Large blue crystals [VOCl_2_(CH_3_CN)_2_(H_2_O)] (**1**) precipitated from this solution. The bottom layer was re-extracted with fresh acetonitrile until it was completely discolored, followed by the separation of crystalline product **1**. As a result, the total yield of the product was 19.3 g (74%). C_4_H_8_Cl_2_N_2_O_2_V (237.97): calcd. C, 20.19; H, 3.39; N, 11.77; found. C 19.98, H 3.31, N 11.74%. IR (Nujol, cm^−1^): 3837 s, 3391 s, 3256 w, 3218 w, 3181 w, 2429 w, 2388 w, 2315 s, 2287 m, 2247 w, 1956 m, 1603 s, 1410 w, 1358 w, 1028 s, 984 s, 945 s, 816 m, 600 s.

### 4.5. Synthesis of 3,5-(CF_3_)_2_C_6_H_3_-mian (**2**)

Acenaphtenequinone (5.0 g, 27.5 mmol), 3,5-trifluoromethylaniline (12.6 g, 55.0 mmol) and acetic acid (1 mL) were mixed in 50 mL of toluene. The reaction mixture was boiled for 24 h with a Dean–Stark trap, then cooled to room temperature and filtered to remove unreacted acenaphthenequinone. To the resulting solution, we added 2 g of magnesium oxide, and the mixture was stirred at room temperature for an hour. Then, the solution was filtered from the precipitate and all volatile components were removed via a rotary evaporator. To the solid residue, 50 mL of acetone was added. The solution was filtered and concentrated to a volume of 20 mL. From the resulting solution, crystalline pastel-yellow precipitate **2** was isolated. Yield 4.6 g (43%). C_20_H_9_F_6_NO (393.28): calcd. C, 61.08; H, 2.31; N, 3.56; found. C 60.68, H 2.35, N 3.24%. IR (KBr, cm^−1^): 3433 br.s, 3068 w, 1732 s, 1656 m, 1594 m, 1449 w, 1381 vs, 1285 vs, 1181 s, 1119 s, 1030 w, 959 w, 945 w, 888 w, 850 w, 831 w, 779 m, 727 w, 703 w, 680 m, 598 w, 530 w, 443 w, 414 w. NMR ^1^H (300 MHz, C_6_D_6_, 25 °C, δ, ppm, *J*/Hz): E-isomer (56%): 7.82 (d, 1H, naphthalene part, *J* = 7.0), 7.59 (s., 1H, C_6_H^o^_2_H^p^(CF_3_)_2_), 7.47 (d, 1H, naphthalene part, *J* = 8.2), 7.45 (d, 1H, naphthalene part, *J* = 8.2), 7.43 (d, 1H, naphthalene part, *J* = 7.2), 7.31 (s., 2H, C_6_H^o^_2_H^p^(CF_3_)_2_), 7.22 (dd, 1H, naphthalene part, *J* = 7.0, 8.2), 7.02 (dd, 1H, naphthalene part, *J* = 7.2, 8.2); Z-isomer (44%): 7.78 (d, 1H, naphthalene part, *J* = 7.1), 7.60 (s., 1H, C_6_H^o^_2_H^p^(CF_3_)_2_), 7.47 (d, 1H, naphthalene part, *J* = 8.2), 7.37 (d, 1H, naphthalene part, *J* = 8.2), 7.21 (d, 1H, naphthalene part, *J* = 8.1), 7.12 (s., 2H, C_6_H^o^_2_H^p^(CF_3_)_2_), 7.08 (dd, 1H, naphthalene part, *J* = 7.0, 8.2), 7.59 (dd, 1H, naphthalene part, *J* = 7.2, 8.1). NMR ^19^F (282.4 MHz, C_6_D_6_, 25 °C, δ, ppm): −62.55, −62.62.

### 4.6. Synthesis of [VOCl_2_(dpp-mian)(CH_3_CN)] (**3**)

#### 4.6.1. Method A

[VCl_3_(THF)_3_] (0.28 g, 1.0 mmol) was added to a solution of dpp-mian (0.34 g, 1.0 mmol) in tetrahydrofuran (20 mL). As a result, the color of the solution changed from orange to brown. Concentrating the solution produced an oily substance. After the complete removal of all volatile components in vacuo, acetonitrile was added to the residue in air. The color of the solution gradually changed from brown to green. After vigorous stirring for half an hour, the resulting solution was concentrated in a vacuum to a volume of 5 mL. One day later, the green crystalline complex **3** was isolated from this solution. The yield was 0.11 g (21%).

#### 4.6.2. Method B

[VOCl_2_(CH_3_CN)_2_(H_2_O)] (0.24 g, 1.0 mmol) was added to a solution of dpp-mian (0.34 g, 1.0 mmol) in acetonitrile (30 mL). As a result, the color of the solution changed from orange to green. After stirring for 30 min, the solution was concentrated by removing volatiles in vacuo to a volume of 5 mL. From the resulting solution, 0.43 g of crystalline green complex **3** was isolated. Yield 83%. C_26_H_26_Cl_2_N_2_O_2_V (520.34): calcd. C, 60.01; H, 5.04; N, 5.38; found. C 60.50, H 5.00, N 5.40%. IR (KBr, cm^−1^): 3400 br.w, 3068 w, 2970 m, 2916 m, 2868 m, 2326 w, 2295 s, 2248 m, 1704 vs, 1622 m, 1601 s, 1584 s, 1489 w, 1467 m, 1433 w, 1381 w, 1359 w. 1330 w, 1291 s, 1222 w, 1174 w, 1101 m, 1041 m, 997 vs, 924 m, 829 s, 804 m, 775 vs, 709 w, 675 w, 632 w, 588 w, 533 m, 472 w.

### 4.7. Synthesis of [{VOCl(3,5-(CF_3_)_2_C_6_H_3_-bian)(H_2_O)}{VOCl_3_(3,5-(CF_3_)_2_C_6_H_3_-bian)}]·2.85DME (**4**)

#### Method B

[VOCl_2_(CH_3_CN)_2_(H_2_O)] (0.24, 1.0 mmol) was added to a solution of 3,5-(CF_3_)_2_C_6_H_3_-mian (0.39 g, 1.0 mmol) in acetonitrile (30 mL). As a result, the color of the solution changed from orange to brown. After stirring for 30 min, the solution was concentrated to a volume of 5 mL. On cooling, a small amount of a light amorphous precipitate of acenaphthenequinone (according to the NMR spectroscopy data) was formed from this solution. The solution was filtered, and all solvent was removed in vacuo. The dry residue was dissolved in dimethoxyethane (5 mL) with slight heating. One day later, violet needle crystals of complex **4** precipitated from the solution at room temperature. The yield was 0.18 g (41%). C_67.39_H_54.47_Cl_4_F_24_N_4_O_8.69_V_2_ (1759.02): calcd. C 46.01, H 3.12, N 3.19; found. C 48.33, H 3.17, N 3.14%. IR (Nujol, cm^−1^): 3381 m(br), 3084 w, 1667 m, 1634 s, 1585 m, 1372 s, 1282 s, 1184 m, 1144 s, 1106 w, 1052 w, 1022 w, 989 m, 978 m, 895 m, 860 m, 849 m, 833 m, 780 m, 727 m, 702 m, 683 m, 648 w, 620 w, 596 w, 583 w.

### 4.8. Synthesis of [VOCl_2_(CH_3_CN)(DME)] (5)

To begin, 2.0 g of [VOCl_2_(CH_3_CN)_2_(H_2_O)] (**1**) was dissolved in 20 mL of 1,2-dimethoxyethane. The resulting solution was filtered from the amorphous precipitate. Then, all volatiles were removed in a vacuum. The resulting residue was dissolved in 5 mL of acetonitrile with heating, and all volatiles were removed again in vacuo. The residue was redissolved in 10 mL of 1,2-dimethoxyethane, and the solution was left at room temperature for 1 day. As a result, 1.94 g of complex **5** was formed as light blue crystals. The yield was 86%. C_6_H_13_Cl_2_NO_3_V (269.02): C, 26.79; H, 4.87; N, 5.21; found. C 26.89, H 5.26, N 5.21%. IR (Nujol, cm^−1^): 3253 w, 3218 w, 3050 w, 3031 w, 2396 m, 2316 s, 2288 m, 2245 s, 2048 w, 1953 m, 1914 w, 1649 m, 1618 w, 1576 w, 1417 m, 1366 w, 1279 s, 1261 s, 1242 s, 1209 w, 1189 s, 1158 m, 1092 vs, 1039 vs, 980 s, 941 m, 874 s, 841 m, 804 s.

### 4.9. Computational Details

The single point calculations based on the experimental X-ray structures of **2** (four disordered CF_3_-groups in the appropriate model structure were corrected by removing formally excess of fluorine atoms), **3** and **4** (experimental geometries were used as-is) and full geometry optimization procedure for Z and E isomers of **2** have been carried out at the DFT level of theory using the dispersion-corrected hybrid functional ωB97XD [87] with the help of Gaussian-09 program package [88]. The 6-31+G* basis sets were used for all atoms. The spin restricted approximation for the model structures with closed electron shells and unrestricted method for the model structures with open electron shells were employed. No symmetry restrictions have been applied during the geometry optimization procedure. The Hessian matrices were calculated analytically for all optimized model structures to prove the location of the correct minimum on the potential energy surface (no imaginary frequencies were found in all cases). The topological analysis of the electron density distribution, conducted with the help of the atoms in molecules (QTAIM) method developed by Bader [62], was performed by using the Multiwfn program (version 3.7) [89]. The molecular orbitals were plotted in the Chemcraft program (http://www.chemcraftprog.com) (accessed on 18 March 2021). The Cartesian atomic coordinates for the model structures are presented in Table S3, Supporting Information.

### 4.10. Alkane Oxygenation Reactions

Pyrex cylindrical vessels, with vigorous stirring of the reaction mixture, were used for the oxidation of alkanes with hydrogen peroxide and were typically carried out in air in a thermostat-monitored solution. The total volume of the reaction solution was 2.5 mL (CAUTION: the combination of air or molecular oxygen and H_2_O_2_ with organic compounds at elevated temperatures may be explosive!). Initially, a portion of 50% aqueous solution of hydrogen peroxide was added in one portion to the solution of the catalyst, co-catalyst (PCA), and substrate in acetonitrile. We used the optimal concentration of the catalyst since, with a decrease in the concentration to 1 × 10^−4^ M, the reaction rate decreases threefold, and an increase in the catalyst concentration to 1 × 10^−3^ M increases the reaction rate insignificantly. A decrease in temperature by 20 degrees leads to the stratification of the reaction mixture, and an increase in temperature increases the amount of over-oxidation products. The temperature of the reaction was 50° C. The aliquots of the reaction solution were analyzed by GC (the instrument was a 3700, fused silica capillary column FFAP/OV-101 20/80 *w*/*w*, 30 m × 0.2 mm × 0.3 µm; helium was used as a carrier gas. The attribution of peaks was made via comparison with chromatograms of authentic samples). Usually, the samples were analyzed twice, i.e., before and after the addition of the excess of solid PPh_3_). This method was developed and used previously [76,77].

Alkyl hydroperoxides are transformed in the GC injector into a mixture of the corresponding ketone and alcohol. For this reason, we quantitatively reduced the reaction samples with PPh_3_ to obtain the corresponding alcohol. This method allowed us to calculate the real concentrations, not only of the hydroperoxide but of the alcohols and ketones present in the solution at a given moment (see Figure S5). In the absence of a catalyst, the reaction proceeds extremely slowly; in five hours, the yield of products was no more than 3–5%. In the oxidation reaction of cyclohexane in the presence of complex **3** as a catalyst, the total yield of cyclohexanol and cyclohexanone was 43% (TON = 400; concentration the sum of products divided by the concentration of the catalyst). For the reaction in the presence of catalyst **4**, the maximum yield of cyclohexanol and cyclohexanone was 27% (TON = 250).

The reactions for the oxidation of alcohols to the corresponding ketones were carried out in a similar way. The reaction samples were treated with triphenylphosphine to reduce the excess of *tert*-butyl hydroperoxide.

Regarding the oxidation of alcohols, for the oxidation of phenyl ethanol, the yield of acetophenone was 80% (TON = 800) for complex **4,** and 50% (TON = 460) in the presence of complex **3**. In the oxidation reaction of cyclohexanol, in the presence of complex **3** as a catalyst, the total yield was 42% (TON = 400). For the oxidation reaction of 2-heptanol in the presence of catalyst **3**, the maximum yield of 2-heptanone was 35% (TON = 300). We calculated the experimental error using the methodology given in the book by [90]. The errors are from 10 to 20% for various experiments.

## Figures and Tables

**Table 1:** Redox potentials ^[a]^ determined by CV for **2–4** and dpp-mian in CH_2_Cl_2_.

Compound	Reduction, *E*_1/2_ (Δ*E* ^[b]^, mV)	Oxidation, *E*_pa_
dpp-mian	−1.16 (160), −1.94 (irrev.)	1.64
**2**	−0.85 (155), −1.67 (irrev.)	-
**3**	−1.23 (irrev.), −1.62 (130)	1.12, 1.62
**4**	−0.98 (irrev.), −1.56 (110)	1.08, 1.52

[a] *E*, V versus Ag/AgCl; the potentials were measured at 100 mVs^−1^; ferrocene was used as an internal standard, and the Fc/Fc^+^ potential was 0.57 V; [b] Δ*E* = |*E*_a_ − *E*_c_|.

**Table 2:** Values of the density of all electrons—ρ(**r**), Laplacian of electron density—∇^2^ρ(**r**) and appropriate λ_2_ eigenvalues, energy density—H_b_, potential energy density—V(**r**), and Lagrangian kinetic energy—G(**r**) (a.u.) at the bond-critical points (3, –1), corresponding to intermolecular stacking interactions in the X-ray structures **2** and **3**, and estimated strength for these contacts E_int_ (kcal/mol).

Contact *	ρ(r)	∇^2^ρ(r)	λ_2_	H_b_	V(r)	G(r)	E_int_ **
**2**							
C18···C46 3.360 Å	0.005	0.016	−0.005	0.001	−0.002	0.003	0.6
C9···C55 3.360 Å	0.005	0.016	−0.005	0.001	−0.002	0.003	0.6
C12···C51 3.398 Å	0.006	0.016	−0.006	0.001	−0.003	0.003	0.9
C14···C49 3.398 Å	0.006	0.016	−0.006	0.001	−0.003	0.003	0.9
**3**							
C12···C78 3.394 Å	0.006	0.017	−0.006	0.001	−0.003	0.004	0.9
C11···C76 3.467 Å	0.006	0.015	−0.006	0.001	−0.003	0.003	0.9
C19···C72 3.491 Å	0.006	0.015	−0.006	0.001	−0.003	0.003	0.9
C15···C70 3.549 Å	0.005	0.013	−0.005	0.001	−0.002	0.003	0.6

* The Bondi’s (shortest) van der Waals radius for carbon atom is 1.70 Å [63]. The numeration of atoms corresponds to their ordering in the attached xyz-files for model structures (Supporting Information). ** E_int_ ≈ −V(**r**)/2 [64].

**Table 3:** Values of the density of all electrons—ρ(**r**), Laplacian value of electron density—∇^2^ρ(**r**) and appropriate λ_2_ eigenvalues, energy density—H_b_, potential energy density—V(**r**), and Lagrangian kinetic energy—G(**r**) (a.u.) at the bond-critical points (3, –1), corresponding to the coordination bond V–O and noncovalent contact V···O in the V–O···V moiety in X-ray structure **4**.

Contact *	ρ(r)	∇^2^ρ(r)	λ_2_	H_b_	V(r)	G(r)
V1–O7 1.640 Å	0.233	0.932	−0.233	−0.141	−0.516	0.375
V2···O7 2.013 Å	0.076	0.446	−0.076	0.005	−0.102	0.107

* The numeration of atoms corresponds to their ordering in the attached xyz-file for model structure.

## Data Availability

The study did not report any data.

## Supplementary Materials

Figure S1: Overlay of two independent molecules A (red) and B (blue) with inversion for complex 3, Figure S2: 1H-NMR spectrum of 2 in C6D6, Figure S3: Distribution of HOMOs and LUMOs in hypothetical active species for oxygenation of alkanes and alcohols in case of 3 (viz. species with H2O2 and OH• ligands), Figure S4: Distribution of HOMOs and LUMOs in hypothetical active species for oxygenation of alkanes and alcohols in case of 4 (viz. species with H2O2 and OH• ligands), Figure S5: Accumulation of cyclohexanol and cyclohexanone in oxidation of cyclohexane (0.46 M) with hydrogen peroxide (2.0 M, 50 % aqueous) catalyzed by compound 3 (5 × 10–4 M) in MeCN at 50 °C before (graph A) and after treating the reaction sample with PPh3 (graph B), Figure S6: UV-vis spectra of 1, 3, 4, dpp-mian and 3,5-(CF3)2C6H3-bian (briefly CF3-bian), Figure S7: Aliphatic region of 1H NMR spectrum of complex 3 (a) and complex 3 with the addition of a free dpp-mian ligand (b) in CD3CN. Squares-coordinated dpp-mian ligand, circles-free dpp-mian ligand, Figure S8: 1H NMR spectrum of complex 4 in acetonitrile before (up) and after the addition (down) of the free 3,5-(CF3)2C6H3-bian ligand. The black circles show new signals that appear on the spectrum after the addition of 3,5-(CF3)2C6H3-bian, Table S1: Selected distances [Å] and angles [°] in complexes 3 and 5, Table S2: Selected distances [A] and angles [°] in complex 4, Table S3: Cartesian atomic coordinates for model structures, Table S4: Crystallographic data and refinement details for 2–5.

## Sample Availability

Samples of the compounds **1**–**5** are available from the authors.
