# Supplementary material for: Novel Oxidovanadium Complexes with Redox-Active R-Mian and R-Bian Ligands: Synthesis, Structure, Redox and Catalytic Properties
Source: Molecules. 2021 Sep 21;26(18):5706. doi: 10.3390/molecules26185706 (PMC8465707; doi:10.3390/molecules26185706)
Supplement: Supplementary file 1 [file molecules-26-05706-s001.zip › molecules-1366045-supplementary.pdf]

Novel Oxidovanadium Complexes with Redox-Active R-Mian and R-Bian Ligands: Synthesis, Structure, Redox and Catalytic Properties

Anton N. Lukoyanov [1], Iakov S. Fomenko [2], Marko I. Gongola [2], Lidia S. Shul'pina [3], Nikolay S. Ikonnikov [3], Georgiy B. Shul'pin [4,5], Sergey Y. Ketkov [1], Georgy K. Fukin [1], Roman V. Rumyantcev [1], Alexander S. Novikov [6], Vladimir A. Nadolinny [2], Maxim N. Sokolov [2] and Artem L. Gushchin [2,*]

1 Razuvaev Institute of Organometallic Chemistry, Russian Academy of Sciences, Tropinina, 49,
603950 Nizhny Novgorod, Russia; anton@iomc.ras.ru (A.N.L.); sketkov@iomc.ras.ru (S.Y.K.); gera@iomc.ras.ru (G.K.F.); romanrum@iomc.ras.ru (R.V.R.)

2 Nikolaev Institute of Inorganic Chemistry, Siberian Branch of Russian Academy of Sciences, 3 Acad. Lavrentiev Ave., 630090 Novosibirsk, Russia; fom1-93@mail.ru (I.S.F.); m.gongola@g.nsu.ru (M.I.G.); spectr@niic.nsc.ru (V.A.N.); caesar@niic.nsc.ru (M.N.S.)

3 Nesmeyanov Institute of Organoelement Compounds, Russian Academy of Sciences, ulitsa Vavilova, dom 28, 119991 Moscow, Russia; shulpina@ineos.ac.ru (L.S.S.); ikonns@ineos.ac.ru (N.S.I.)

4 Semenov Federal Research Center for Chemical Physics, Russian Academy of Sciences, ulitsa Kosygina 4, 119991 Moscow, Russia; gbsh@mail.ru

5 Chair of Chemistry and Physics, Plekhanov Russian University of Economics, Stremyannyi pereulok,
dom 36, 117997 Moscow, Russia

6 Institute of Chemistry, Saint Petersburg State University, Universitetskaya Nab., 7/9, 199034 Saint Petersburg, Russia; a.s.novikov@spbu.ru *

* Correspondence: gushchin@niic.nsc.ru

Figure S1. Overlay of two independent molecules **A** (red) and **B** (blue) with inversion for complex **3**.

Figure S2. ^{1}H-NMR spectrum of **2** in C_6D_6.

Figure S3. Distribution of HOMOs and LUMOs in hypothetical active species for oxygenation of alkanes and alcohols in case of **3** (viz. species with H_2O_2 and $OH^•$ ligands).

Figure S4. Distribution of HOMOs and LUMOs in hypothetical active species for oxygenation of alkanes and alcohols in case of **4** (viz. species with H_2O_2 and $OH^•$ ligands).

Figure S5. Accumulation of cyclohexanol and cyclohexanone in oxidation of cyclohexane (0.46 M) with hydrogen peroxide (2.0 M, 50 % aqueous) catalyzed by compound **3** (5×10^{-4} M) in MeCN at 50 °C before (graph A) and after treating the reaction sample with PPh_3 (graph B).

Figure S6. UV-vis spectra of **1**, **3**, **4**, dpp-mian and 3,5-$(CF_3)_2C_6H_3$-bian (briefly CF_3-bian).

Figure S7. Aliphatic region of ^{1}H NMR spectrum of complex **3** (a) and complex **3** with the addition of a free dpp-mian ligand (b) in CD_3CN. Squares - coordinated dpp-mian ligand, circles - free dpp-mian ligand.

Figure S8. ^{1}H NMR spectrum of complex **4** in acetonitrile before (up) and after the addition (down) of the free 3,5-$(CF_3)_2C_6H_3$-bian ligand. The black circles show new signals that appear on the spectrum after the addition of 3,5-$(CF_3)_2C_6H_3$-bian.

Table S1. Selected distances [Å] and angles [°] in complexes **3** and **5**.

Table S2. Selected distances [A] and angles [°] in complex **4**.

Table S3. Cartesian atomic coordinates for model structures.

Table S4. Crystallographic data and refinement details for **2-5**.

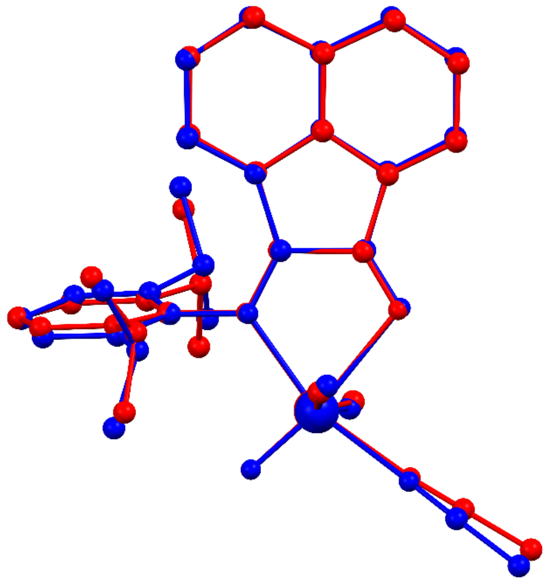

Figure S1. Overlay of two independent molecules **A** (red) and **B** (blue) with inversion for complex **3**.

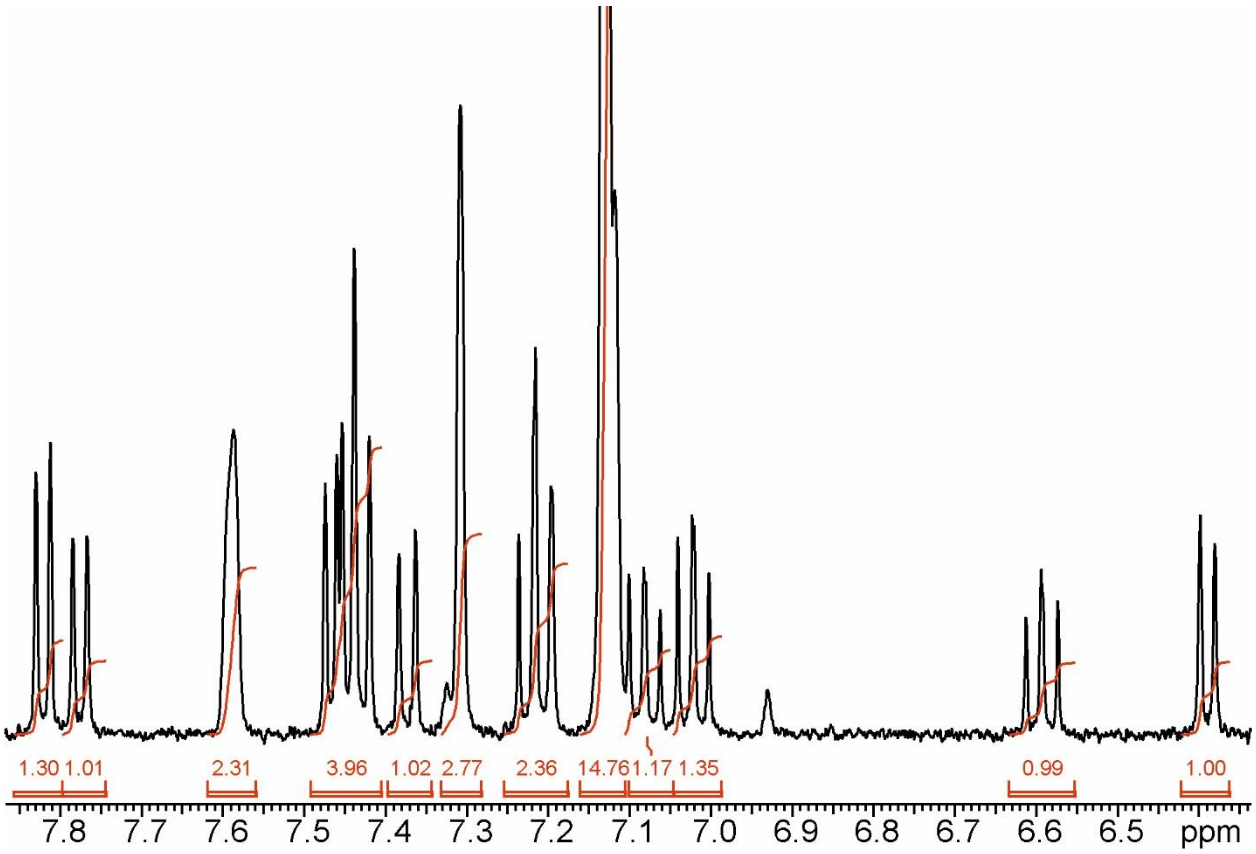

Figure S2. ^{1}H-NMR spectrum of **2** in C_6D_6.

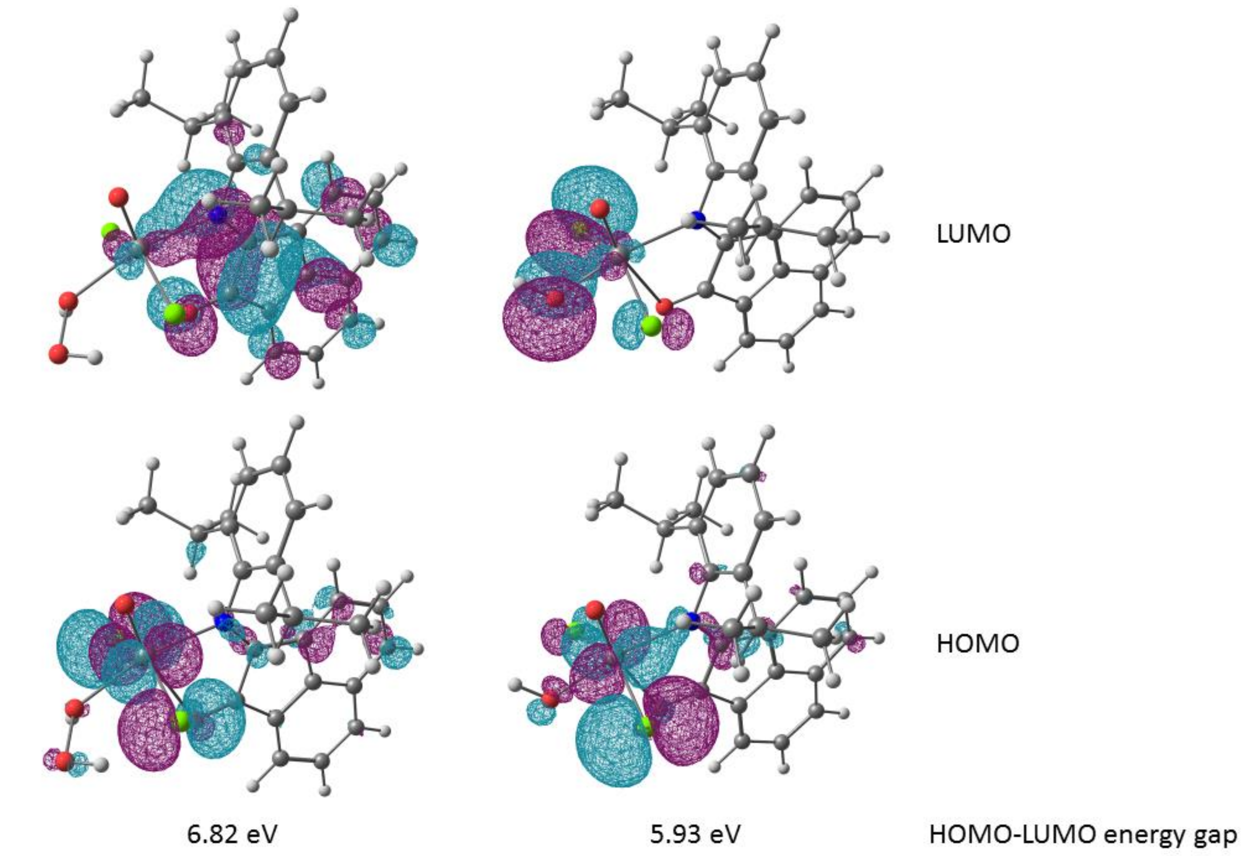

Figure S3. Distribution of HOMOs and LUMOs in hypothetical active species for oxygenation of alkanes and alcohols in case of **3** (viz. species with H_2O_2 and $OH^{\bullet}$ ligands).

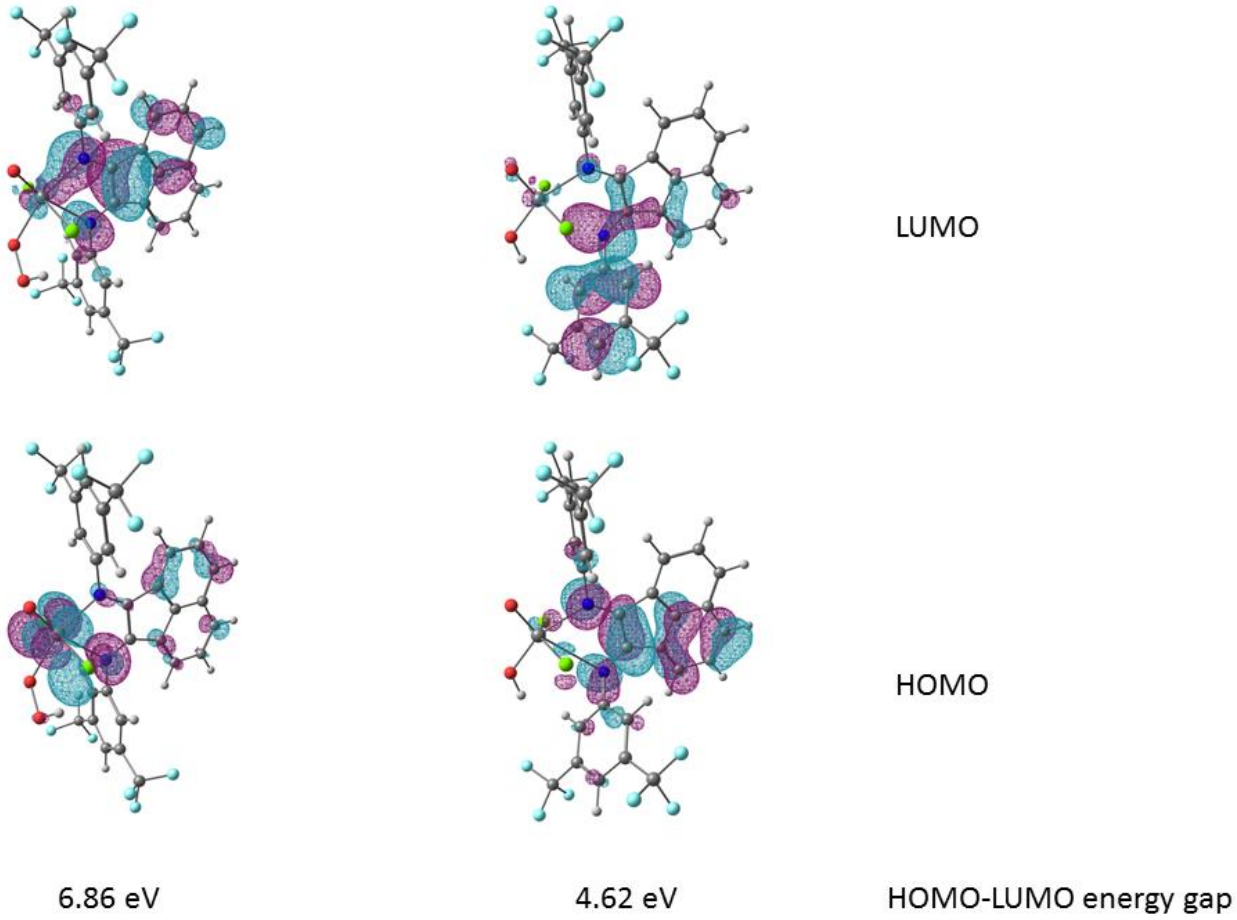

Figure S4. Distribution of HOMOs and LUMOs in hypothetical active species for oxygenation of alkanes and alcohols in case of **4** (viz. species with H_2O_2 and $OH^{\bullet}$ ligands).

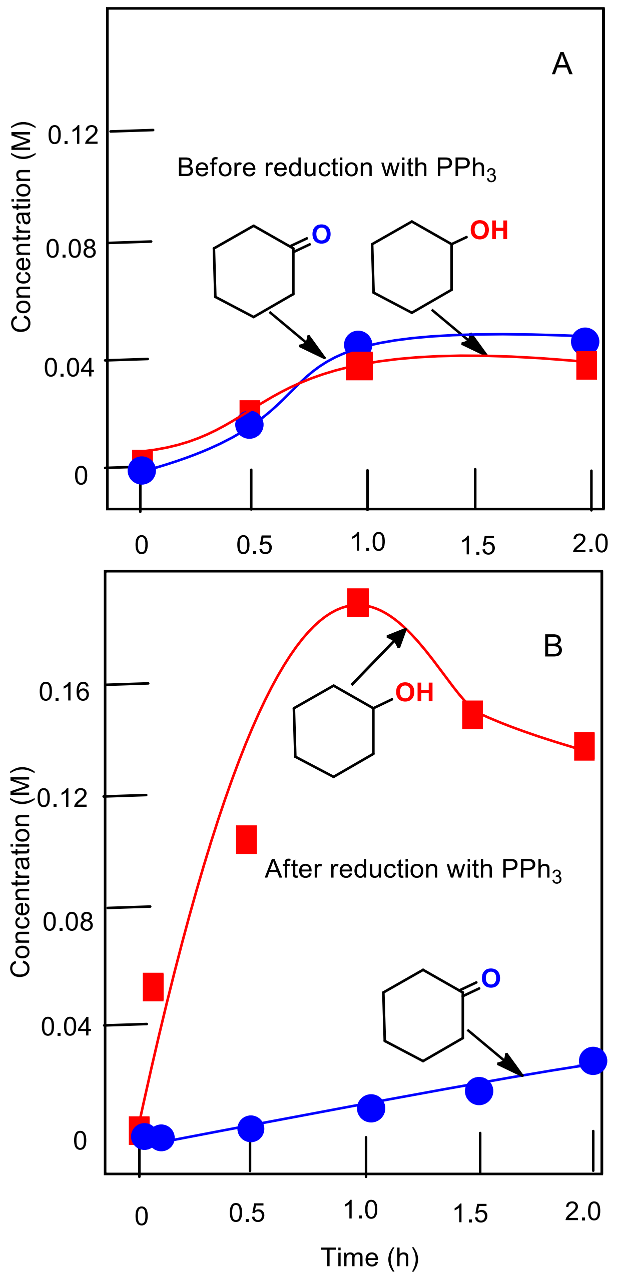

Figure S5. Accumulation of cyclohexanol and cyclohexanone in oxidation of cyclohexane (0.46 M) with hydrogen peroxide (2.0 M, 50 % aqueous) catalyzed by compound **3** (5×10^{-4} M) in MeCN at 50 °C before (graph A) and after treating the reaction sample with PPh_3 (graph B).

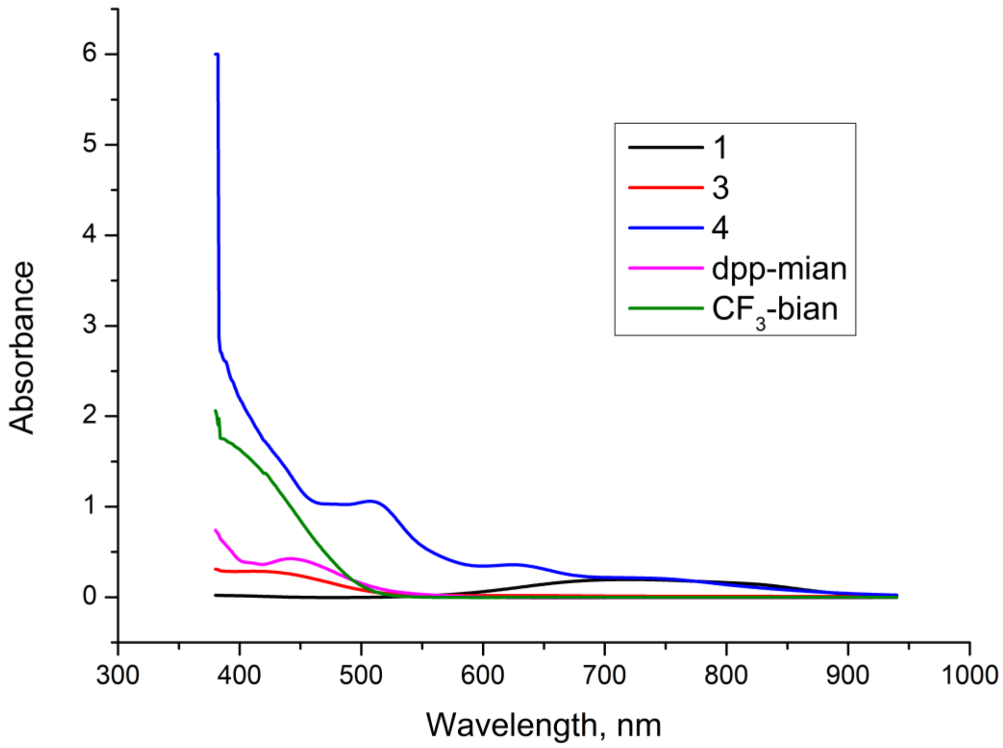

Figure S6. UV-vis spectra of **1**, **3**, **4**, dpp-mian and 3,5-$(CF_3)_2C_6H_3$-bian (briefly CF_3-bian).

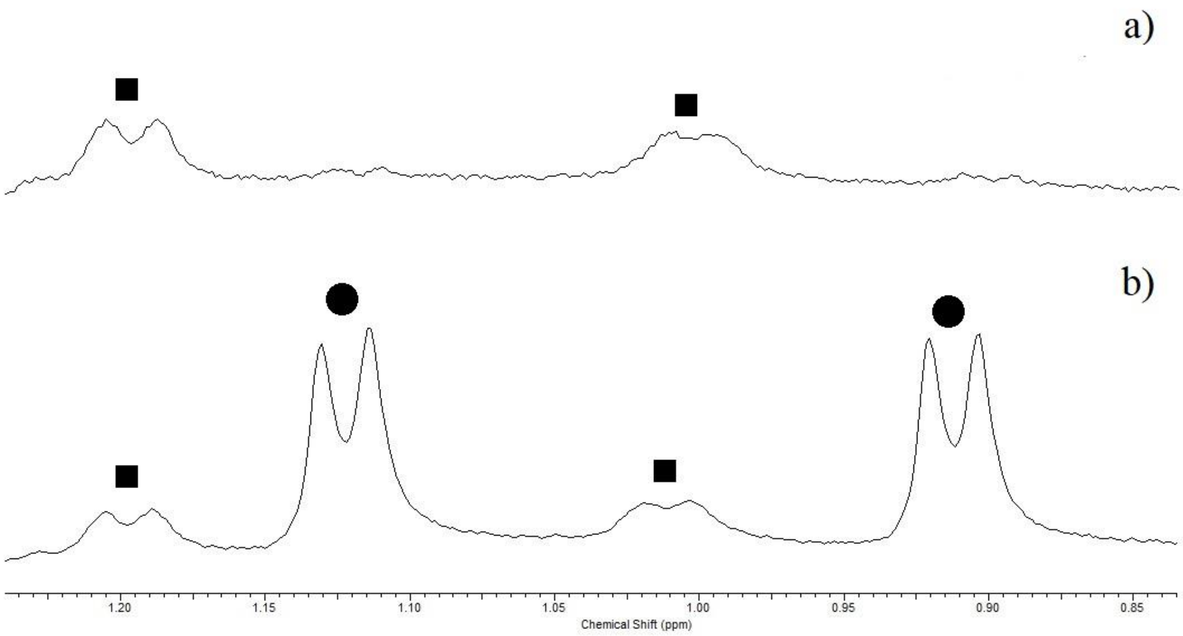

Figure S7. Aliphatic region of ^{1}H NMR spectrum of complex **3** (a) and complex **3** with the addition of a free dpp-mian ligand (b) in CD_3CN. Squares - coordinated dpp-mian ligand, circles - free dpp-mian ligand.

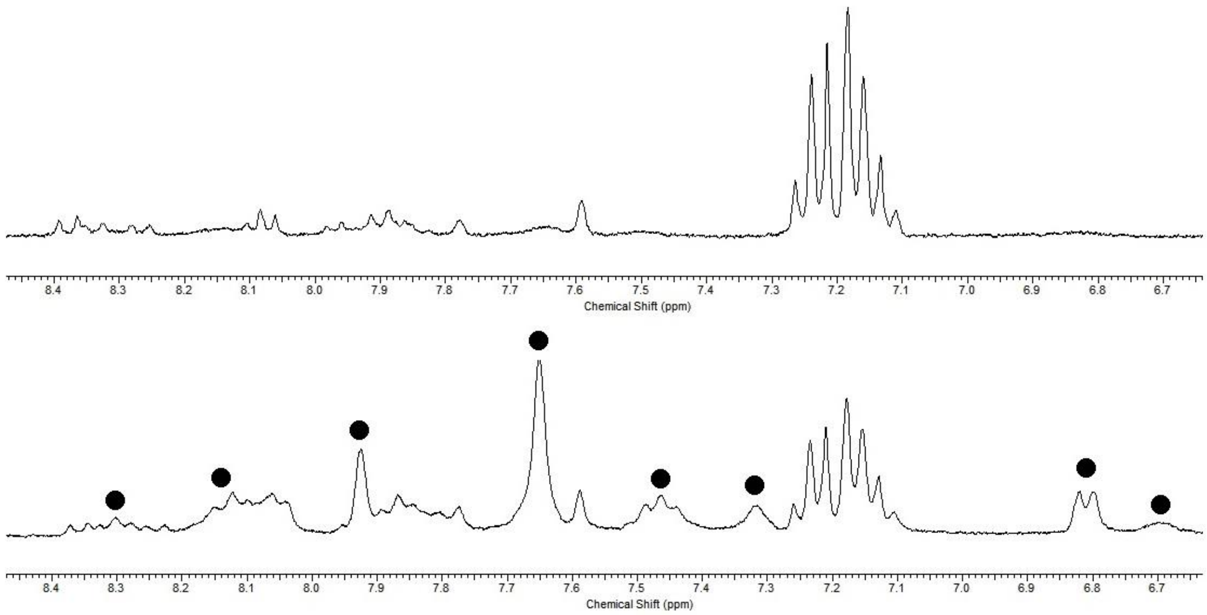

Figure S8. ^{1}H NMR spectrum of complex **4** in acetonitrile before (up) and after the addition (down) of the free 3,5-$(CF_3)_2C_6H_3$-bian ligand. The black circles show new signals that appear on the spectrum after the addition of 3,5-$(CF_3)_2C_6H_3$-bian.

Table S1. Selected distances [Å] and angles [°] in complexes **3** and **5**.

Distance [Å] / Angle [°]	**3**		**5**	Angle [°]	**3**		**5**
	**A**	**B**			**A**	**B**	
V(1)-O(1)	2.339(2)	2.387(2)	1.5913(9)	O(1)-V(1)-Cl(1)	78.89(6)	77.86(5)	99.56(3)
V(1)-O(2)	1.585(2)	1.585(2)	2.2873(8)	O(1)-V(1)-Cl(2)	80.51(6)	80.61(6)	103.03(3)
V(1)-N(1)	2.163(2)	2.160(2)	2.1277(10)	N(1)-V(1)-O(2)	96.46(10)	96.14(9)	77.85(3)
V(1)-N(2)/O(3)	2.107(3)	2.107(2)	2.1112(8)	N(1)-V(1)-N(2)/O(3)	160.90(10)	162.62(9)	85.56(4)
V(1)-Cl(1)	2.3588(9)	2.3669(8)	2.3453(3)	N(1)-V(1)-Cl(1)	90.44(6)	90.30(6)	167.02(3)
V(1)-Cl(2)	2.3243(10)	2.3146(8)	2.3169(3)	N(1)-V(1)-Cl(2)	90.67(6)	90.64(6)	90.91(3)
O(1)-C(1)	1.218(3)	1.218(3)	-	O(2)-V(1)-N(2)/O(3)	102.59(11)	101.09(10)	73.56(3)
N(1)-C(2)/C(1)	1.282(3)	1.282(3)	1.1400(16)	O(2)-V(1)-Cl(1)	99.37(10)	100.37(8)	89.72(2)
C(1)-C(2)	1.525(3)	1.526(4)	1.4530(16)	O(2)-V(1)-Cl(2)	101.95(10)	102.00(8)	88.06(2)
O(1)-V(1)-N(1)	75.39(8)	74.57(8)	91.89(4)	N(2)/O(3)-V(1)-Cl(1)	85.15(9)	84.45(7)	87.35(2)
O(1)-V(1)-O(2)	171.58(9)	170.45(9)	165.11(4)	N(2)/O(3)-V(1)-Cl(2)	86.79(10)	87.98(8)	161.62(3)
O(1)-V(1)-N(2)/O(3)	85.53(9)	88.13(9)	95.12(4)	Cl(1)-V(1)-Cl(2)	158.38(3)	157.38(3)	92.419(12)

Table S2. Selected distances [A] and angles [°] in complex **4**.

Bond	Distance [Å]	Bond	Distance [Å]
V(1)-O(1)	1.6409(13)	N(1)-C(1)	1.277(2)
V(1)-N(1)	2.2702(16)	N(2)-C(2)	1.279(2)
V(1)-N(2)	2.1433(16)	N(3)-C(29)	1.290(2)
V(1)-Cl(1)	2.3665(6)	N(4)-C(30)	1.280(2)
V(1)-Cl(2)	2.3285(6)	C(1)-C(2)	1.506(2)
V(1)-Cl(3)	2.2902(5)		
V(2)-O(1)	2.0125(13)	Angle	Angle [°]
V(2)-O(2)	2.0046(15)	N(1)-V(1)-N(2)	73.76(6)
V(2)-O(3)	1.5882(14)	Cl(1)-V(1)-Cl(2)	163.78(2)
V(2)-N(3)	2.1266(16)	Cl(1)-V(1)-Cl(3)	93.98(2)
V(2)-N(4)	2.3651(16)	N(3)-V(2)-N(4)	73.18(6)
V(2)-Cl(4)	2.3251(5)	V(1)-O(1)-V(2)	169.77(9)
C(29)-C(30)	1.514(3)		

Table S3. Cartesian atomic coordinates for model structures.

Atom	X	Y	Z
**2**			
O	2.497146	1.872128	6.912885
N	3.708566	1.860579	4.317862
F	8.578400	1.466530	0.883667
F	8.567155	1.357614	3.069321
F	8.016353	-0.344175	1.893542
F	2.510330	2.303837	-0.602273
F	4.179977	1.336692	-1.509948
F	4.273382	3.420696	-1.232562
C	3.129128	2.804093	6.486548
C	3.811730	2.853422	5.100739
C	4.470881	4.164191	5.003245
C	4.204975	4.846079	6.219512
C	3.433354	4.096999	7.137193
C	3.088867	4.645646	8.344639
H	2.581621	4.165409	8.958633
C	3.520812	5.952119	8.632797
H	3.299742	6.329949	9.453327
C	4.257701	6.684814	7.740441
H	4.516138	7.549801	7.963866
C	4.633581	6.150047	6.483780
C	5.366498	6.788259	5.457307
H	5.673483	7.657651	5.579032
C	5.624620	6.134910	4.291244
H	6.111943	6.575858	3.633243
C	5.188817	4.828198	4.038656
H	5.382991	4.415321	3.228265
C	4.427985	1.856408	3.093830
C	5.773882	1.502641	3.095807
H	6.205068	1.321282	3.899594
C	6.468471	1.420670	1.909046
C	5.846360	1.691121	0.705911
H	6.321503	1.638313	-0.091798
C	4.509039	2.041237	0.701586
C	3.794517	2.113516	1.879501
H	2.891072	2.333578	1.863180
C	7.896177	1.004071	1.949257
C	3.840122	2.331444	-0.592280
O	1.891114	8.715686	6.270862
N	0.679694	8.727236	8.865885
F	-4.163401	9.555152	12.407936
F	-4.183445	8.721210	10.442701
F	-3.654880	10.846152	10.801655
F	1.877929	8.283977	13.786020

F	0.208282	9.251123	14.693695
F	0.114877	7.167119	14.416309
C	1.259132	7.783722	6.697198
C	0.576529	7.734392	8.083008
C	-0.082622	6.423623	8.180502
C	0.183285	5.741736	6.964235
C	0.954905	6.490815	6.046554
C	1.299392	5.942168	4.839107
H	1.806639	6.422406	4.225114
C	0.867447	4.635696	4.550950
H	1.088517	4.257865	3.730420
C	0.130559	3.903000	5.443305
H	-0.127879	3.038013	5.219880
C	-0.245321	4.437767	6.699967
C	-0.978238	3.799556	7.726440
H	-1.285223	2.930164	7.604715
C	-1.236361	4.452905	8.892503
H	-1.723683	4.011956	9.550504
C	-0.800558	5.759616	9.145091
H	-0.994732	6.172493	9.955482
C	-0.039725	8.731406	10.089917
C	-1.385622	9.085173	10.087939
H	-1.816809	9.266532	9.284153
C	-2.080212	9.167144	11.274701
C	-1.458101	8.896693	12.477836
H	-1.933244	8.949501	13.275545
C	-0.120779	8.546578	12.482161
C	0.593742	8.474299	11.304246
H	1.497187	8.254237	11.320567
C	-3.507918	9.583743	11.234490
C	0.548138	8.256370	13.776027
**3**			
V	7.482962	7.183395	15.958786
Cl	8.779585	5.504839	14.908314
Cl	5.575698	8.220845	16.759174
O	5.835572	5.970986	14.725560
O	8.631236	8.100942	16.546106
N	7.291659	8.228612	14.077511
N	7.269562	5.764005	17.501267
C	5.757282	6.436905	13.599974
C	6.566023	7.653579	13.182656
C	6.278221	7.918178	11.775711
C	5.305292	6.941120	11.408920
C	4.949467	6.056878	12.437561
C	4.031590	5.056654	12.213695
H	3.778230	4.454801	12.903687
C	3.477695	4.960413	10.907876

H	2.838892	4.280532	10.728430
C	3.835078	5.815540	9.901227
H	3.435355	5.716526	9.045110
C	4.784355	6.842316	10.104355
C	5.286512	7.770537	9.156438
H	4.951346	7.761316	8.267573
C	6.246803	8.678040	9.506586
H	6.585700	9.262523	8.838746
C	6.756952	8.783386	10.821134
H	7.413198	9.434918	11.038734
C	7.868251	9.522722	13.765954
C	7.027962	10.646007	13.704366
C	7.633152	11.877640	13.448641
H	7.091216	12.657110	13.412979
C	8.981870	11.999419	13.247524
H	9.367863	12.852690	13.087898
C	9.776393	10.869497	13.278916
H	10.708841	10.954240	13.117891
C	9.246197	9.613779	13.539843
C	5.513023	10.606447	13.874893
H	5.231031	9.652076	13.973047
C	5.065221	11.373463	15.121236
H	5.542045	11.029553	15.905301
H	4.100594	11.256008	15.248027
H	5.266294	12.325959	15.008435
C	4.834708	11.187926	12.618115
H	5.108843	10.672578	11.830931
H	5.102926	12.123761	12.505548
H	3.861263	11.136984	12.719140
C	10.138034	8.386698	13.452867
H	9.583204	7.583833	13.670963
C	11.288867	8.456329	14.459750
H	10.925879	8.557467	15.364325
H	11.860820	9.223862	14.249582
H	11.816901	7.632192	14.409908
C	10.670218	8.222892	12.028725
H	9.918776	8.197316	11.400147
H	11.178725	7.387660	11.964062
H	11.254132	8.978699	11.809231
C	7.272750	4.940538	18.276005
C	7.233339	3.870529	19.267599
H	7.480119	3.022442	18.843068
H	6.328066	3.800018	19.636314
H	7.865370	4.070605	19.989289
V	4.119140	2.642453	5.748932
Cl	2.779011	4.339451	6.690568
Cl	6.030679	1.601350	4.935078

O	5.742907	4.029840	6.699268
O	2.982156	1.623674	5.321951
N	4.468768	1.807355	7.714093
N	4.245625	3.916115	4.076556
C	5.947783	3.687307	7.854671
C	5.255373	2.483047	8.468428
C	5.695322	2.397825	9.863263
C	6.590251	3.491574	10.043700
C	6.811579	4.263506	8.885149
C	7.689802	5.318671	8.910405
H	7.853965	5.838170	8.132155
C	8.340095	5.606818	10.127192
H	8.967006	6.319909	10.158540
C	8.091314	4.882594	11.272073
H	8.533115	5.121407	12.078465
C	7.191960	3.790797	11.272612
C	6.849262	2.951299	12.359752
H	7.212572	3.122604	13.220665
C	5.994864	1.890358	12.178689
H	5.788483	1.342087	12.926553
C	5.409325	1.577438	10.931981
H	4.836916	0.825629	10.833813
C	3.895258	0.569830	8.232116
C	2.596434	0.563268	8.738960
C	2.070454	-0.642809	9.176725
H	1.187383	-0.666357	9.526217
C	2.801659	-1.804486	9.114845
H	2.415419	-2.625543	9.396205
C	4.100613	-1.776021	8.642278
H	4.606754	-2.579785	8.624571
C	4.683388	-0.592288	8.191076
C	1.775335	1.832281	8.908029
H	2.366849	2.612918	8.706300
C	0.599198	1.861664	7.925514
H	0.936987	1.780976	7.009120
H	0.114319	2.707857	8.021628
H	-0.005680	1.114925	8.117625
C	1.277176	1.975140	10.345944
H	2.033956	1.894662	10.963359
H	0.623530	1.270352	10.536743
H	0.854643	2.852036	10.459662
C	6.104899	-0.649714	7.691052
H	6.387017	0.278277	7.447642
C	6.204560	-1.525291	6.433021
H	5.563190	-1.208736	5.763054
H	6.001767	-2.455463	6.665501
H	7.112315	-1.470293	6.067819

C	7.066230	-1.174915	8.764741
H	7.052780	-0.572929	9.537934
H	7.974249	-1.215890	8.398387
H	6.785840	-2.071495	9.043885
C	4.314078	4.698473	3.252675
C	4.441284	5.672184	2.197088
H	3.660855	6.264873	2.203602
H	4.495119	5.212425	1.333304
H	5.254357	6.201133	2.336835
**4**			
V	5.442738	7.228730	5.032129
V	1.884612	7.085341	4.283145
Cl	5.818117	6.297893	2.888686
Cl	5.662100	8.413089	7.024891
Cl	5.825206	5.257036	6.131596
Cl	-0.205814	6.241269	3.714325
O	3.815974	7.254474	4.826305
O	2.616667	6.572779	2.489694
H	3.449149	6.449921	2.364778
H	2.205714	6.364816	1.852725
O	1.565499	8.639191	4.198498
N	7.691428	7.504335	4.890283
N	5.757189	9.133788	4.102284
N	1.576664	6.977882	6.384757
N	2.352497	4.860404	4.932730
F	9.668935	2.397977	4.391194
F	11.561155	2.984311	3.959493
F	9.789292	3.524849	2.720748
F	12.003064	5.802931	8.357837
F	10.144087	6.662715	9.154116
F	10.393834	4.601011	9.044547
F	1.620821	9.843519	0.384795
F	3.603841	10.120474	-0.309825
F	2.341229	11.811247	0.068642
F	2.728137	12.463355	6.311841
F	1.876998	13.538738	4.665598
F	0.928302	11.761315	5.394755
F	-2.739354	9.421766	7.275545
F	-1.941360	11.298358	6.647307
F	-2.212920	10.913343	8.739838
F	4.037156	10.811468	8.599204
F	2.523870	12.103915	9.364686
F	3.016462	10.301083	10.409112
F	-0.373801	2.213897	1.674041
F	0.637286	0.347388	1.961287
F	1.063804	1.541710	0.249380
F	5.908307	0.954702	3.467718

F	5.837540	1.452869	1.401835
F	6.555306	2.878176	2.818709
C	8.060723	8.611493	4.378497
C	6.959393	9.540196	3.939585
C	7.587358	10.739471	3.380133
C	8.986445	10.505919	3.468807
C	9.331884	9.253488	4.043586
C	10.652329	8.882303	4.110297
H	10.902796	8.041758	4.475368
C	11.628569	9.777574	3.624377
H	12.544238	9.529039	3.672268
C	11.297697	11.000194	3.082758
H	11.985605	11.576486	2.771064
C	9.934628	11.412490	2.983791
C	9.437605	12.617122	2.428941
H	10.044225	13.271558	2.103029
C	8.083555	12.846585	2.358450
H	7.776431	13.663853	1.983948
C	7.128475	11.906196	2.825098
H	6.197210	12.080270	2.754853
C	8.664757	6.537653	5.303287
C	9.018871	5.509712	4.445392
H	8.634996	5.453256	3.578252
C	9.941098	4.563516	4.868094
C	10.495189	4.618741	6.142136
H	11.124677	3.966842	6.427204
C	10.106250	5.650671	6.989224
C	9.183870	6.610272	6.586349
H	8.914799	7.302181	7.179105
C	10.323407	3.462381	3.929826
C	10.680448	5.722885	8.379594
C	4.655239	9.948100	3.673850
C	4.250711	9.886022	2.348116
H	4.702754	9.324978	1.728938
C	3.170286	10.661837	1.944912
C	2.499222	11.464597	2.846137
H	1.764365	11.995438	2.562076
C	2.910747	11.487249	4.173022
C	3.985755	10.729993	4.599052
H	4.259560	10.745893	5.508597
C	2.688990	10.607984	0.521135
C	2.123189	12.308408	5.137198
C	2.011873	5.945736	7.021727
C	2.462973	4.751313	6.203135
C	2.936066	3.736856	7.155683
C	2.761631	4.309084	8.437409
C	2.194890	5.610368	8.431533

C	1.941071	6.244716	9.622551
H	1.542118	7.106727	9.638515
C	2.288717	5.582483	10.830458
H	2.115471	6.013972	11.658890
C	2.870041	4.332316	10.836251
H	3.100968	3.925740	11.663205
C	3.126253	3.648919	9.626537
C	3.716025	2.370450	9.475508
H	3.986070	1.884438	10.245797
C	3.903182	1.822309	8.224476
H	4.313828	0.968715	8.152013
C	3.501383	2.494046	7.043998
H	3.621487	2.090953	6.192164
C	1.202243	8.166982	7.098197
C	-0.107550	8.621036	7.025055
H	-0.761704	8.133861	6.537985
C	-0.444285	9.799751	7.676448
C	0.508953	10.528022	8.380889
H	0.267912	11.328415	8.832313
C	1.814800	10.067982	8.413823
C	2.178131	8.899366	7.767936
H	3.080103	8.601426	7.781455
C	-1.840952	10.348182	7.591862
C	2.846427	10.825862	9.194754
C	2.666052	3.800571	4.024191
C	1.619420	3.251690	3.284206
H	0.724253	3.530683	3.436941
C	1.901882	2.293365	2.322475
C	3.209648	1.891045	2.066623
H	3.396548	1.246086	1.394603
C	4.231222	2.451254	2.812648
C	3.979881	3.412042	3.788083
H	4.693742	3.795620	4.283823
C	0.804349	1.615862	1.558309
C	5.629698	1.953138	2.612186
Optimized equilibrium structure of Z isomer of **2**			
C	5.216472	0.632807	-2.278072
C	3.799300	0.651767	-2.339590
C	3.099747	0.304362	-1.209945
C	3.793270	-0.059401	-0.036294
C	2.941767	-0.396954	1.040176
C	3.488366	-0.775371	2.240040
C	4.904013	-0.814148	2.352982
C	5.897297	0.276300	-1.128999
C	5.192385	-0.089175	0.049659
C	5.736782	-0.484519	1.302320
C	1.552954	-0.242375	0.570394

C	1.647854	0.209038	-0.919245
O	0.711546	0.408654	-1.656507
N	0.517149	-0.467338	1.268532
C	-0.784127	-0.262709	0.769703
C	-1.568536	-1.365677	0.436363
C	-2.878968	-1.173747	0.011074
C	-3.430446	0.100827	-0.062430
C	-2.644788	1.190532	0.302473
C	-1.332067	1.019614	0.723220
C	-3.247134	2.571397	0.297559
F	-3.852630	2.849218	1.473119
F	-2.322035	3.529406	0.102507
F	-4.179654	2.713696	-0.661879
C	-3.694658	-2.364783	-0.421136
F	-5.014839	-2.162517	-0.256469
F	-3.372284	-3.476539	0.267564
F	-3.502470	-2.647423	-1.727067
H	5.781883	0.906227	-3.163917
H	3.286689	0.932599	-3.254665
H	2.858369	-1.040710	3.083630
H	5.342722	-1.114254	3.299897
H	6.984442	0.273595	-1.125813
H	6.815088	-0.529173	1.431277
H	-1.145301	-2.362084	0.505828
H	-4.453882	0.243525	-0.389401
H	-0.724111	1.873131	1.004007
Optimized equilibrium structure of E isomer of **2**			
C	6.103214	-0.028303	0.234367
C	5.379068	-0.201594	-0.972614
C	4.007839	-0.184338	-0.912618
C	3.354381	0.002044	0.323486
C	1.938083	-0.002528	0.229658
C	1.198413	0.166937	1.377996
C	1.881707	0.341414	2.611006
C	5.468107	0.152683	1.447704
C	4.049421	0.173247	1.529879
C	3.257571	0.346761	2.697414
C	1.609630	-0.211160	-1.201875
C	2.959071	-0.335051	-1.952023
O	3.086864	-0.515655	-3.137514
N	0.514379	-0.294963	-1.838407
C	-0.706533	-0.171383	-1.155877
C	-1.377909	-1.316214	-0.718476
C	-2.598043	-1.188414	-0.066070
C	-3.176017	0.062404	0.141334
C	-2.510416	1.190879	-0.323544
C	-1.284885	1.084771	-0.975507

C	-3.085482	2.561051	-0.070013
F	-2.530707	3.126959	1.025277
F	-2.859498	3.400449	-1.096263
F	-4.412709	2.526907	0.137686
C	-3.347029	-2.419954	0.376239
F	-3.970297	-2.228512	1.555086
F	-2.537448	-3.483899	0.520901
F	-4.297518	-2.764988	-0.515744
H	7.188547	-0.039779	0.201053
H	5.895695	-0.344367	-1.916895
H	0.113934	0.172341	1.368526
H	1.293150	0.475528	3.513456
H	6.056622	0.280891	2.352595
H	3.741154	0.484053	3.661068
H	-0.931388	-2.290843	-0.882959
H	-4.130798	0.152497	0.646547
H	-0.770037	1.967906	-1.339713

Table S4. Crystallographic data and refinement details for **2-5**.

	**2**	**3**	**4**	**5**
Formula	$C_{20}H_9F_6NO$	$C_{26}H_{26}Cl_2N_2O_2V$	$C_{67.39}H_{54.47}Cl_4F_{24}$	$C_6H_{13}Cl_2NO_3V$
M_r [g mol^{-1}]	393.28	520.33	1759.10	269.01
T [K]	298(2)	100(2)	100(2)	100(2)
crystal system	Triclinic	Monoclinic	Triclinic	Monoclinic
space group	P-1	$P2_1$	P-1	P2(1)/c
a [Å]	8.1566(11)	8.9310(2)	13.1908(5)	7.7496(3)
b [Å]	8.6540(14)	19.1295(5)	13.3831(5)	22.3917(8)
c [Å]	13.489(2)	14.6532(4)	21.0312(8)	6.9171(2)
α [°]	78.052(13)	90	97.7180(10)	90
β [°]	87.131(12)	95.9709(12)	99.0640(10)	113.2700(10)
γ [°]	64.591(14)	90	93.5660(10)	90
V [Å^3]	840.6(2)	2489.85(11)	3619.9(2)	1102.66(7)
Z	2	4	2	4
ρ_{calc}, [g cm^{-3}]	1.554	1.388	1.614	1.620
μ [mm^{-1}]	0.142	0.639	0.526	1.360
F(000)	396	1076	1773	548
crystal size, [mm]	0.46 × 0.17 × 0.09	0.22 × 0.06 × 0.05	0.42 × 0.29 × 0.16	0.36 × 0.30 × 0.10
$\theta_{min}/\theta_{max}$ [°]	3.090 - 28.498	2.528 - 29.574	2.291 - 30.508	2.861 - 30.000
index ranges	$-10 \le h \le 10$<br>$-11 \le k \le 11$	$-12 \le h \le 12$<br>$-26 \le k \le 26$	$-18 \le h \le 18$<br>$-19 \le k \le 19$	$-10 \le h \le 10$<br>$-31 \le k \le 31$
reflections collected/ unique	13145/4186	33926/13985	51367 / 22054	16359/3215
R_{int}	0.0406	0.0319	0.0340	0.0290
data/restraints / parameters	4186 / 147 / 297	13985 / 1 / 606	22054 / 27 / 1058	3215 / 0 / 124
S	1.037	1.014	1.021	1.048
final *R* indices [I>2σ(I)]	$R_1 = 0.0712$<br>$wR_2 = 0.1576$	$R_1 = 0.0346$<br>$wR_2 = 0.0771$	$R_1 = 0.0458$<br>$wR_2 = 0.1015$	$R_1 = 0.0229$<br>$wR_2 = 0.0591$
R indices (all data)	$R_1 = 0.1444$<br>$wR_2 = 0.1841$	$R_1 = 0.0406$<br>$wR_2 = 0.0796$	$R_1 = 0.0744$<br>$wR_2 = 0.1127$	$R_1 = 0.0260$<br>$wR_2 = 0.0602$
peak/hole [e Å^{-3}]	0.307 / -0.420	0.489 / -0.474	0.749 / -0.854	0.322 / -0.550
